# Arginine: II. Interactions of Its Salt Bridges with Branched Aliphatic Side Chains

**DOI:** 10.1021/acs.jpcb.5c02172

**Published:** 2025-07-09

**Authors:** Christopher M. Ng, Vivian Kui, Katherine Y. Han, Eric R. Kempson, Margaret Mandziuk

**Affiliations:** † Department of Chemistry, 5894 New York University, New York, New York 10003, United States; ‡ Current address: The Fu Foundation School of Engineering, Columbia University, New York, New York 10027, United States

## Abstract

Previous analyses of the structures deposited in the PDB revealed that arginine (Arg) side chains are often in close contact with leucine (Leu) side chains. In the previous paper, we studied interactions between Leu and Arg side chains alone [Ng et al. https://doi.org/10.1021/acs.jpcb.5c02168]. In this work, we have focused on the interactions between Leu side chains and salt bridges formed between Arg and acidic side chains. We inspected the trimer structures of the three side chains: Leu, Arg, and an acidic residue in the high-resolution files in the PDB. We also performed optimization of the model trimers with the ωB97X-D density functional and aug-cc-pVTZ basis set. We found that the salt bridges in a relatively low-polarity environment are most likely in a dynamic equilibrium between an ionic form and two neutral tautomers. That leads to the increased distances between Arg and the acidic residue as well as to the increased density of low-energy vibrational states, consequently increasing specific heat and entropy. The Leu residue controls the polarity of the environment. These findings explain why an increased number of salt bridges provides increased stability to the thermophilic enzymes and increases the fluctuations and mobility of psychrophilic proteins. Further studies are needed to find out whether methyl protons are scrambled with amino protons. We also performed calculations on the trimers with the doubly protonated Arg side chain. Energy of such trimers is lower than the energy of the monoprotonated trimers. Their lowest energy is obtained after a proton transfer to an acidic residue, and the guanidinium ion returns to the lowest energy planar structure. Such proton transfer may occur in membrane proteins where salt bridges are interspersed between nonpolar residues.

## Graphical abstract

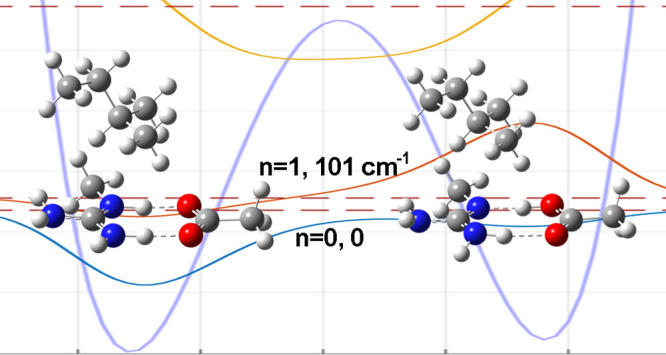

## Introduction

Arg amino acid (AA) plays key roles in many biological processes.^1,2^ It is important for catalysis.^3^ Arg-rich peptides are able to penetrate cell membranes.^4^ Commonly occurring mutations in the tumor suppressor p53 protein involve Arg mutations.^5^ Arg side chain is terminated with a guanidino (Gdn) group. Due to the very high p*K*_a_ of GdnH^+^, it is assumed that the guanidine moiety is protonated even at internal positions in proteins.^6,7^

In the PDB search for close contact pairs between Leu and Arg side chains, we found that Arg in such close contact pairs is often involved in a salt bridge (SB).^8^ Our criterion for the selection of a close contact pair was that at least one distance between an amino nitrogen of Arg and a carbon of Leu is less than 3.5 Å. In our previous paper, we focused on Arg·Leu close contact pairs where Arg was not forming an SB.^8^ Our calculations at the ωB97X-D/aug-cc-pVTZ level^9,10^ indicated an attractive interaction between a model of Arg side chain, methyl guanidinium ion (mGdnH+), and a model of Leu, 2-methylbutane (TMB). Depending on the relative orientation of the monomers, the interaction energy (absolute value) reached up to 8.9 kcal/mol. In the complex, TMB acted as a base and transferred a small amount of electronic charge to mGdnH^+^. Close contacts between hydrogen atoms of TMB and mGdnH^+^ resembled dihydrogen bonding with distances smaller than the sum of their van der Waals radii (2.2–2.4 Å). We suggested that hydrogen exchange may occur between methyl and amino groups, as observed in some dihydrogen-bonded complexes.^11^

The distances between nitrogen atoms of mGdnH^+^ and carbon atoms of TMB methyl groups, optimized in our calculations, were longer than some of the distances found in the PDB structures. In order to decrease these distances, we doubly protonated mGdn at an amino group that is in close contact with the methyl groups of TMB. This increased the interaction energy (absolute value) and stabilized pairs up to 26.3 kcal/mol. Distances between hydrogen atoms decreased, as well. In several cases, they became shorter than 2 Å. Energy of such extra protonated species is lower than the energy of complexes with the monoprotonated mGdnH^+^.^8^ Even if the structures with the doubly protonated Arg side chain are not stable, they may play a role as intermediates in proton transfer.

Arg side chains can form salt bridges with the terminal carboxyl group (OXT) or head groups of aspartic acid (Asp), or glutamic acid (Glu),^12^ as well as multiple hydrogen bonds with polar AA, or backbones. Hydrogen bonds between oppositely charged groups, such as the side chains of Arg and Asp or Arg and Glu, are “charge enhanced.” They are stronger than hydrogen bonds between neutral groups^13^ and were given the special name of salt bridges (SBs). In computational studies, the Arg side chain is often represented by a methyl guanidinium ion (mGdnH^+^)^14–16^ and Asp or Glu side chains by an acetate ion (Ac^–^).^14,15,17^ The possible modes of SB formation between mGdnH^+^ and Ac^–^ are represented in Figure 1a. Side-on and back-on types of SB can form bidentate and monodentate SB, while the back-side type can form only monodentate SB.

**Figure 1:**
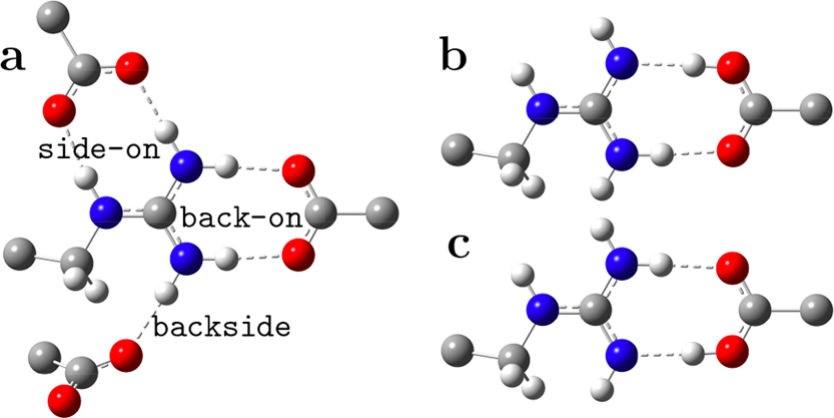
Various ways of forming an SB between mGdnH^+^ and acetate (a); two tautomeric structures in the back-on arrangement (b,c).

In a low polarity environment, Arg and acidic side chains may be in their neutral tautomeric forms, methyl guanidine (mGdn) and acetic acid (HAc).^14^ Such tautomers for the back-on binding mode are shown in Figure 1b,c. Neutral tautomers are also possible for the side-on mode.

The majority of SBs are in the interior of proteins.^12^ There are computational studies indicating that buried or partially buried salt bridges are destabilizing relative to hydrophobic side chains. However, more recent studies show that in the majority of proteins, SBs are stabilizing.^18^ SBs also form on the surfaces of proteins. In the 2D-IR spectroscopic experiments, Huerta-Viga et al. presented evidence of the formation of intra- and intermolecular SBs in peptides in water.^15^ The SBs on the protein surface are thought to contribute to the thermal stability of proteins.^19^ Thermophilic proteins usually have more SBs on their surface, relative to their mesophilic homologues.^20^

In this paper, we focus on the trimers in which the Leu side chain is in close contact with models of Arg·Glu, Arg·Asp, or Arg·OXT SBs. Based on the analysis of the selected structures from the PDB, our calculations, and the available literature on thermostable proteins, we propose how the presence of Leu near an SB contributes to the enhanced protein thermostability or to the enhanced fluctuations of psychrophilic proteins. We also show how the second, extra protonation of the Arg side chain, supported by Leu, may be involved in proton transfer through an SB.

## Methods

### PDB Search and Analysis

The selection of the PDB files and their parsing has been described in Paper I.^8^ Briefly, we selected the file on the PISCES server containing a list of PDB files culled for the high resolution of 1 Å or better, 15% sequence identity, and an R factor of 0.2.^21^ The PISCES file contained 270 protein chains. These protein chains were downloaded from the PDB on February 8, 2024. They were parsed with a Python script for close contact pairs with distances between the nitrogen atoms of Arg side chains and carbon atoms of Leu below 3.5 Å. When the oxygen atoms of OXT or the acidic side chains of Glu or Asp were closer than 3.5 Å from the nitrogen atoms of the Arg side chain, such pairs were marked as forming an SB. We inspected the structures of these trimers (Leu-Arg-Asp, Leu-Arg-Glu, or Leu-Arg-OXT). The analyses and the molecular graphics were performed with UCSF Chimera.^22^

### Computational Details

As commonly used in calculations, methylguanidine, mGdn, and methyl guanidinium ion, mGdnH^+^, were representing Arg side chains, while acetic acid, HAc, and acetate ion, Ac^–^, modeled the side chains of Asp, Glu, and OXT.^12,15^ 2-Methylbutane, TMB, was used as the model of the Leu residue.

For optimization of energies and structures, we employed the ωB97X-D density functional.^9^ Proper accounting for dispersion is important when interactions involve aliphatic species. This functional accounts for dispersion interactions and has been used with success in calculations of weakly bound complexes.^23–26^ The aug-cc-pVTZ basis set^10^ was used in all calculations. This basis set was found to perform very well with the ωB97X-D functional.^23^ It was already used with the ωB97X-D functional for calculations involving dihydrogen-bonded complexes.^25^

Counterpoise correction^27^ was applied only in some cases, as obtaining the exact electronic energy was not the objective of this work. In the optimizations involving just an SB pair, we also used the MP2/aug-cc-pVTZ method for the comparison with the results obtained with the density functional. We also performed calculations with the polarizable continuum model (PCM)^28^ in chloroform. Chloroform, with a dielectric constant of 4.9, was chosen as its dielectric constant is slightly higher than the average dielectric constant in the interior of proteins, ε = 3.23.^29^ All calculations were performed with the Gaussian16 suite of programs.^30^

## Results

### PDB Structures

All 51 identified structures with Arg·Leu side chains in close contact, in which Arg forms an SB with an acidic residue or an OXT, are listed in Table S1. Among these structures, we found 19 structures with a bidentate type of binding. Both back-on and side-on SBs are present. Five structures with networks of charged side chains contain bidentate SBs. We found that 9 out of 19 structures with bidentate SBs are buried inside proteins, while the remaining 10 are on the proteins’ surfaces. That is in contrast with the statement of Donald et al. that the majority of SBs are found in protein interiors.^12^ Our sample, however, is too small to draw conclusions on whether or not this difference is due to the presence of Leu and Arg side chains in a close approach.

While searching for close N···O contacts, we did not impose constraints on the angles. Even in high-resolution structures, positions of hydrogens are not always available. We monitored the donor–acceptor-acceptor antecedent (DAAA) angle, which for our SBs is represented by the C–O···N angle. The average DAAA angle between side chain residues in proteins was determined by Tan et al. to be 125°. We observed a wide range of DAAA angles between 90° and 150° in our selected structures. Even in bidentate structures with two N···O distances nearly equal, the planes of the guanidinium moiety and carboxylate group were not coplanar. The planes of these groups are tilted. Tan et al. explain this behavior by the tendency to increase the distance between negatively charged nitrogen and oxygen atoms and bring closer together the carboxylate carbon and the nitrogen of the amino group.^31^

In all of the bidentate SB structures, Leu approaches SB with only one methyl group. This methyl group is usually in close contact with one of the NH_2_ groups involved in an SB. A few examples of such structures are presented in Figure S1a–d. In only two cases, the methyl group of Leu is approximately in the plane of the guanidinium ion. In the remaining trimers, the methyl is at an angle below or above the SB plane. In only four structures, Leu is in a close approach to an NH_2_ (or NH−), away from the SB.

The N···O distances in nearly all bidentate SB structures are longer than 2.75 Å. Only in one case (PDB ID 1GKM), one of the distances is lower, at 2.67 Å.

32 structures, identified by us, have monodentate SB binding. Among them, there are 14 backside orientations in which strong interaction between the second N···O pair is not possible. The remaining structures with the monodentate SBs involve back-on or side-on binding modes. In these structures, the formation of a second close contact between Arg and acidic side chains is usually prevented by the formation of hydrogen bonds to neighboring polar side chains or an interaction with water.

In the majority of structures with the monodentate SB, Leu closely approaches the guanidinium ion with one methyl group only, as observed for the bidentate SBs. In only a few structures, Leu approaches the guanidinium ion with more carbon atoms in a parallel-like arrangement. Two representative structures with the monodentate SBs are shown in Figure S1e,f.

An interesting Leu42-Arg292-Glu39 trimer interaction is present in the crystal structure of methionine adenosyltransferase 2A (PDB ID 7RWG). In this file, Arg292 is present at two alternative locations, while its neighbors have just one fixed position. In the structure, the methyl group of Leu42 approaches the amino group of Arg292, involved in an SB, from the side at an angle (see Figure 2). In addition to the formation of an SB, the guanidinium ion of Arg292 forms hydrogen bonds with tyrosine (Tyr296) and threonine (Thr374). The amino group that is in close contact with Leu42 forms a hydrogen bond with the backbone carbonyl of lysine (Lys285). The carbonyl of phenylalanine (Phe282) is 3.1 Å above this amino group. Nitrogen atoms of guanidinium are close to the carbon atoms of tilted Tyr371.

**Figure 2:**
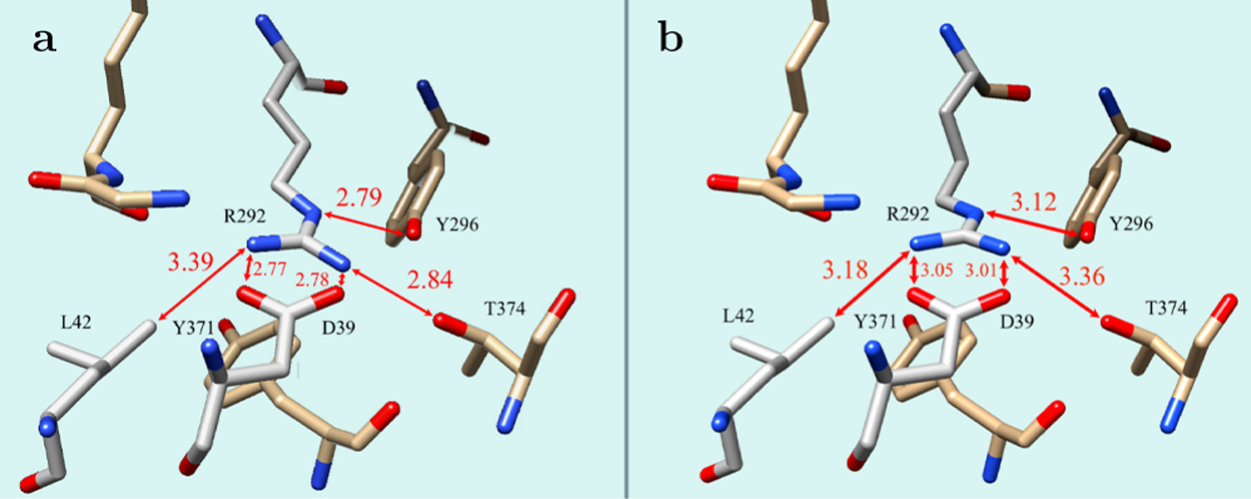
Two alternative locations of Arg292 in the structure of methionine adenosyltransferase 2A (PDB ID 7RWG). Other residues in the figure have only one location. Arg292 forms SB with Glu39. (a) With the larger separation of Leu and Arg side chains, at 3.39 Å (lower left corner), the distances In the SB are shorter, at 2.77 and 2.79 Å; (b) When methyl of Leu42 approaches the amino group closer, at 3.18 Å, the distances in the SB are elongated to 3.05 and 3.01 Å.

In the more abundant form (Arg292 alternative location A, with a 0.65 fraction), the N···O distances in the SB are 2.77 and 2.79 Å. In this structure, the methyl carbon of Leu42 is 3.39 Å from the nitrogen atom of the amino group of Arg292. With Arg292 in alternative location B, the N···O distances in SB are significantly elongated to 3.01 and 3.05 Å. In this case, the methyl carbon of Leu gets much closer to the guanidinium nitrogen at 3.18 Å.

What is the reason for the increased distance in an SB? Is the interaction with Leu important for the shift of the guanidinium ion and the decrease of the N···C distance to 3.18 Å, or is the distance shortening just due to the interactions with other residues while Leu is immobilized in the crowded environment around NH_2_? Further studies are needed to explain why Leu and Arg side chains are able to approach each other so closely with distances below the sum of their van der Waals radii (3.25 Å).

### Computations

#### Interaction of TMB with mGdnH^+^ in an SB

In this work, we focus on the Leu side chain (TMB) interaction with the bidentate SBs. We found several local minimum energy structures for our model of three interacting side chains (TMB· mGdnH^+^·Ac^–^). Energies of some of our optimized structures are listed in Table 1. These structures were optimized with three-fragment-counterpoise corrections.^27,32^ The interaction energy (absolute value) of the trimers is high, more than 130 kcal/mol, due to the presence of SBs. In order to evaluate the contribution of TMB to the interaction in the trimer, we calculated the interaction energy between an SB and TMB in the geometry optimized with counterpoise correction for the three monomers. We also optimized structures with two fragments, separating the TMB from the SB. These interaction energies are also included in Table 1. The interaction energy of the TMB with SB differs by no more than 0.03 kcal/mol in these two methods of calculation. It is lower by about 2 kcal/mol relative to that calculated for TMB and mGdnH^+^ alone.^8^

**Table 1:** Energy, *E*, of the TMB·mGdnH^+^·Ac^–^ Structures Optimized with the Counterpoise Correction for the Three Monomers^a^

str.	*E* (E_h_)	Δ*E*_*int*_ (kcal/mol)			N···C	H···H	N_1_···O_1_	N_2_···O_2_
		3-fragments	TMB-SB @3-frag. geom.	TMB-SB 2-frag. optim.	(Å)			
**T1**	–671.628769	–135.79	–7.02	–6.99	3.475	2.432	2.608	2.635
**T2**	–671.627475	–135.23	–6.14	–6.13	3.461	2.527	2.610	2.627
**T3**	–671.626711	–135.23	–6.26	–6.25	3.373	2.375	2.633	2.606
**T4**	–671.626544	–134.37	–5.55	–5.54	3.517	2.527	2.628	2.616
**T5**	–671.624400	–133.83	–4.91	–4.90	3.542	2.215	2.599	2.620
**T6**	–671.623236	–132.44	–3.32	–3.32	3.467	2.556	2.608	2.621
**T7**	–671.623125	–132.53	–3.98	–3.97	3.616	2.295	2.604	2.621
**T8**	–671.621616	–132.50	–3.03	–3.03	3.352	2.295	2.594	2.616

a Interaction energy of the three monomers, Δ**E**_*int*_ (3-fragments). Interaction energy between the TMB and the SB, obtained with the counterpoise correction for two fragments, using the geometry optimized for the three fragments (TMB·SB @3-frag. geom.). Δ**E**_*int*_ (TMB·SB 2-frag. optim.) was optimized with the counterpoise correction for two fragments, TMB and the SB. Also shown are the closest distance between a nitrogen atom of the mGdnH^+^ and a carbon atom of the TMB, N···C, the closest distance between hydrogens of TMB and mGdnH^+^, H···H, and the distances between nitrogen atoms and oxygen atoms in an SB.

All of the structures included in Table 1 are presented in Figure S2. The structures with the lowest energy have the largest area of TMB contact with an SB, maximizing dispersion interactions. Among the bidentate SBs in the reviewed PDB structures, we did not find Leu side chains directly above or below the center of an SB, maximizing the dispersion interactions. It is possible that such structures are present when the distance between the carbons of Leu and the nitrogens of Arg is further apart than our cutoff distance of 3.5 Å. Also, interactions with other neighbors, stronger than dispersion, that are not included in our calculations, may restrict the TMB from approaching an SB center with more atoms. Unhindered rotation of methyl groups in a nonpolar electrostatic field close to SBs, or an exchange of protons between methyl and amino groups that would increase entropy, however, should also be considered as the reason why only one methyl group of Leu closely approaches the amino group involved in a SB of Arg side chain.

In Figure S2, we also display density surfaces with mapped electrostatic potential (ESP). For comparison with the structures of TMB·mGdnH^+^ pairs without an SB, which were analyzed in Paper I,^8^ electron density is drawn at 0.007 e^–^/bohr^3^ and the ESP range is between −0.325 and 0.325 au ESP. The lack of strong red or blue color on Ac^–^ and mGdnH^+^, respectively, indicates that these residues partly neutralize each other. Especially in the central region of SBs, the electrostatic potential is close to zero. In the structures with an SB, the charge of mGdnH^+^ is decreased due to elongated N–H bonds and protons shifted toward the oxygen atoms of Ac^–^. The decreased charge of the guanidinium ion in an SB indicates that dispersion becomes the most important part of the computed interaction energy with Leu and justifies the energetic order of the reported clusters in Table 1.

All of our structures, optimized in a vacuum, have rather short distances between the nitrogen atoms of mGdnH^+^ and the oxygen atoms of Ac^–^. These distances, around 2.6 Å, are much shorter than those observed in the PDB structures reviewed earlier.

In order to analyze how the structures, especially the separations of monomers in SBs, are affected by an environment with a higher dielectric constant, we selected three structures, **T2**, **T5**, and **T6**, for further calculations. In these structures, TMB (Leu methyl group) approaches the amino group of Arg in a different way and should be sufficient to observe the behavior of the trimers in different polarity environments. They are displayed in Figure 3. In the **T2** structure, the methyl guanidinium·acetate pair is in the side-on arrangement, with TMB above their quasi-plane. In the structure **T5**, SB is in the back-on arrangement, and TMB approaches amino groups at an angle, from the side. In **T6**, SB is in the side-on configuration, while TMB approaches an amino group not involved in the SB. The numbering of nitrogen and oxygen atoms in ionic structures is displayed in Figure 3. Energies of the neutral tautomeric structures of **T2**, **T5**, and **T6** are presented in Table S2. The atom numbers in the tautomeric structures are the same. In the lowest energy structure, **T1**, the ethyl branch of TMB is above the SB. It was not selected for further studies as **T1** is more appropriate for representing the interaction of isoleucine (Ile) with an SB. In this work, we concentrated on the interaction of Leu with SBs.

**Figure 3:**
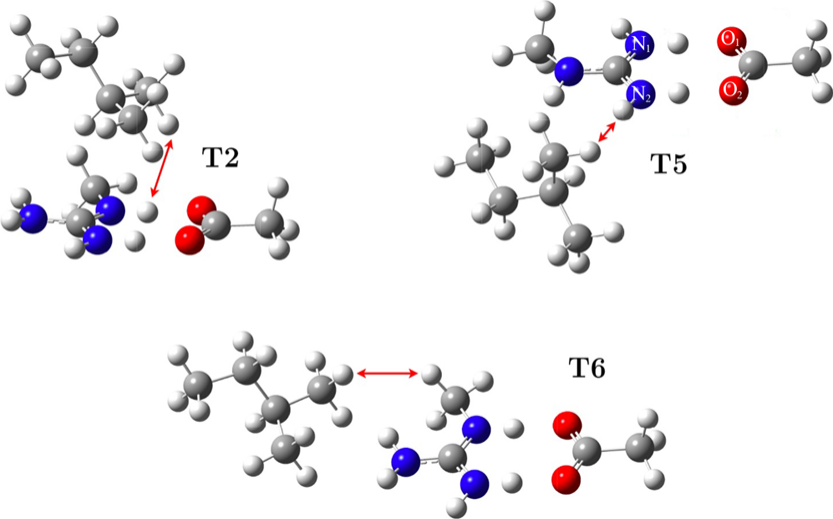
Three optimized SB structures were selected for further studies with labels of nitrogen and oxygen atoms forming a SB (or a hydrogen bond between neutral tautomers). The red arrows indicate the closest contact between hydrogen atoms in each of the structures. The distance is shown in Table 3.

#### Comparison of Structures in Vacuum and in Chloroform

The optimized structures of **T2**, **T5**, and **T6** in vacuum and in chloroform are displayed in Figure S3. In addition to the ionic forms, we also optimized the tautomeric structures with neutral mGua and HAc. Nagy and Erhardt have shown that in a low-polarity environment, below ε = 5, minima exist for these neutral complexes as well.^14^ In our calculations for structures in a vacuum, the ionic structures of **T2** and **T6** have lower energy than those of the structures of neutral tautomers. For **T2**, the gap between the ionic and neutral structures is larger than for **T6**. Note that the interaction energy of **T2** is larger than that of **T6** (see Table 1). For **T5**, the neutral structure N_1_···H–O_1_ has the lowest energy. In this case, SB is in a back-on arrangement.

In order to confirm that the trends observed in our calculations are independent of the choice of density functional, we optimized SB dimers (side-on and back-on) with the MP2 method, as well. Comparison of the energies of SB structures optimized at the ωB97X-D/aug-cc-pVTZ and the MP2/aug-cc-pVTZ levels is presented in Table S3. Interestingly, in a vacuum, both methods favor neutral structures for the dimers. The differences between the energies of the neutral and the ionic structures are even greater when optimized with the MP2 method. This trend changes with an increased dielectric constant. In chloroform, the energy of the neutral tautomeric structures is much higher relative to that of an ionic form for all three types of structures. Although there are energy differences between the optimized structures obtained with the ωB97X-D/aug-cc-pVTZ and the MP2/aug-cc-pVTZ methods, the trends are similar. This is sufficient for us to accept the results from the density functional calculations, as we seek qualitative comparisons.

To what extent does the presence of the Leu residue affect an SB? In a vacuum, the comparison of the results presented in Figure S3, for the interacting three side chains, and in Table S3 for the SB alone, shows that the presence of TMB changes the relative order of energies of ionic and neutral forms. Neutral trimers with Leu side chains have relative energies higher than those calculated just for the hydrogen-bonded dimer structures. The presence of TMB simulates an increased polar environment, although rather small, relative to chloroform. The relative increase of the energy of the neutral structures in the presence of TMB is the highest for **T2**, more than 250 cm^–1^. **T2** has the largest overlap with the SB and the largest interaction energy between TMB and the SB (see Table 1). For **T5** and **T6**, the increase of neutral structures’ energies is smaller and decreases as the interaction energy between TMB and the SB decreases. Note that **T5** has the back-on SB arrangement, where neutral tautomers of dimers have energy much lower than that of the ionic form. In chloroform, the relative energy of neutral complexes increases by approximately 1600–1700 wavenumbers, relative to the energy obtained in a vacuum, independently of the structure type.

The distances between amino nitrogen atoms and carbonyl oxygen atoms in the SBs increase from approximately 2.6 Å in a vacuum to about 2.7 Å in chloroform. The neutral structures have uneven N···O distances. The hydrogen bond with a hydrogen atom close to an oxygen atom is elongated to 2.84–2.88 Å in a vacuum, and by a few hundred Å more in chloroform. The hydrogen bond with a H close to the amino group is shortened in chloroform to values below 2.6 Å.

N···O distances in the structures optimized with the MP2 method are even shorter than those optimized with the ωB97X-D functional (see Table S3). The N···O distances obtained for the optimized structures of the trimers (Figure S3) are very close to the values obtained for the dimers, in a vacuum as well as in chloroform. The N···O distances in SBs optimized in a water environment (dielectric constant, ε = 78) increase further, but only to about 2.75 Å. These results indicate that a polar environment is not sufficient to explain the elongation of the N–O distances in SBs between Arg and acidic side chains, observed in the PDB structures.

#### Comparison of Structures with Increasing Distance between SB Monomers

To what extent does vibrational motion cause an elongation of distances in an SB? We performed a constrained optimization of structure **T2**, with a stepwise increased distance between central carbon atoms of mGdnH^+^ and Ac^–^. Such calculations were also performed for the tautomeric neutral structures. In addition, the optimization of **T2** and its tautomers was performed in chloroform, in order to compare the behavior in a vacuum and in a more polar environment. The selected distances between the central carbon atoms for the structures in a vacuum were 3.85 Å, which is close to the distance in the minimum energy ionic pair structure, 4.00 Å, which is close to the distance in the minimum energy structures of neutral pairs, and additionally at 4.25 Å. In chloroform, where monomers are farther apart, **T2** was optimized with longer C···C distance constraints at 4.00, 4.25, and 4.50 Å. The results are presented in Figure S4.

At a 3.85 Å separation between the carbon atoms, the ionic form is favored. With the increased distance to 4.0 Å, both N–H bond lengths in the ionic structures decrease, while the distance between nitrogen and oxygen atoms increases. At that separation, the energy of the SB is increased by 484.6 cm^–1^. The energies of the neutral structures go down toward their minimum values. The energy of the (N_2_···H–O_2_) structure, constrained at 4.0 Å, is only 198.2 cm^–1^ above the energy of the ionic structure at 3.85 Å (close to the global minimum). At the C···C separation of 4.00 Å, the N···O distances in the SB are 2.71 and 2.74 Å, while the uneven distances in the neutral tautomers are around 2.6 and 2.9 Å.

When the separation between central carbon atoms is 4.25 Å, the energies of the neutral structures are approximately 2000 cm^–1^ below the energy of the ionic structure. The lower energy structure (N_1_···H–O_1_) is 850 cm^–1^ above the global minimum. At this separation of carbon atoms, one of the N···O distances in the neutral structure with the hydrogen atom shifted to oxygen is increased to more than 3 Å, while the shorter one is approximately 2.8 Å.

In the higher polarity environment of chloroform (ε = 4.9), ionic structures are much more stabilized. In addition to increasing the dipole moment by stretching the distance between monomers in an SB, N–H bonds involved in the SB become shorter in chloroform relative to those in a vacuum. Localization of opposite-sign charges increases the dipole moment and strengthens the interactions with the polar environment. With the increased distance between central carbon atoms, the energies of the neutral structures become closer to those of the ionic forms. For the 4.25 Å separation, however, they are still above 1100 cm^–1^. The results of our calculations in the polar environment are consistent with the previous theoretical studies of SBs by Nagy and Erhardt, showing that at ε = 5 and above, ionic SB formation dominates.^14^ They, however, do not consider a lower polarity environment. Recent determination of the average dielectric constant in proteins yields the value of ε = 3.23.^29^ The dielectric constant may be locally lower in the nonpolar interior of proteins.

We found that in a low-polarity environment, there are relatively small energy differences between the ionic SB structure and the neutral tautomers when the monomers are at a different separation. This indicates that these structures may be in a dynamic equilibrium involving tunnelling. Tunneling will be accompanied by fluctuations of the SB distances as well as fluctuations of electronic and protonic charges. Longer N···O distances observed in the crystal structures can be explained by averaging the distances of the ionic structure with the longer distances in neutral tautomers. For the experimentally observed N···O distances that are nearly equal, averaging through tunneling between the neutral structures, in addition to tunneling between the ionic and neutral structures, must be considered. For pairs in which the N···O distances are significantly different, neutral monomers should dominate the structure. Nagy and Erhardt mentioned possible proton relocation to the mGdn·HAc complex; however, for ε = 5, that was not needed.^14^

#### Tunneling between Ionic and Neutral Structures

Is the tunneling of protons between ionic and neutral tautomers feasible? In a formic acid dimer (FAD), the delocalization of protons and tunnelling splitting was demonstrated experimentally.^33–36^ The barrier for a double proton transfer in a FAD has been estimated to be around 2900 cm^–1^.^37,38^ Quantum tunnelling is important for biological systems even at 300 K.^39–41^

The potential energy surface (PES) for the transfer of protons between mGdnH^+^ and Ac^–^, with the minimum energy present at the ionic form and two uneven minima for neutral tautomers, is more complex than that for a FAD, where only two equal energy minima have to be considered. Nevertheless, the transfer of protons in a FAD involves large amplitude fluctuations of O···O distances as well as other intramolecular degrees of freedom. In the path integral calculations of tunnelling splittings in the FAD, Richardson observed coupling of all the degrees of freedom with the transfer of protons.^42^

In the mGdnH^+^ and Ac^–^ complexes, the transfer of protons would involve large amplitude fluctuations of N···O distances, accompanied by the rocking motion of guanidine and acidic acid moieties. Fluctuations would also include intramolecular variation in C–N and C–O bond distances as well as the H–N–H angles of amino groups. In protein interiors, these fluctuations will induce the fluctuations of the neighboring groups.

As a test for feasibility, we performed 1-D quantum mechanical dynamics calculations for the transfer of one proton from the nitrogen atom of mGdnH^+^ to the oxygen atom of Ac^–^. Allowing proton transfer without a N···O distance constraint leads to a large elongation of the intermolecular distance for which the potential is much softer. That is why we held the N···O distances constant at 2.75 Å. The energy path was calculated for the structure **T2**, and involved the transfer of a proton from the N_1_ of the ionic structure (N_1_–H···O_1_), to the O_1_ of the neutral structure (N_1_···H–O_1_). The second proton position was not constrained; its distance to N_2_ varied from 1.06 Å at the minimum energy of the ionic structure to 1.02 Å at the minimum energy of the neutral form. The details of the dynamics calculations are described in the Supporting Information. The results of the calculations are presented in Figure 4.

**Figure 4:**
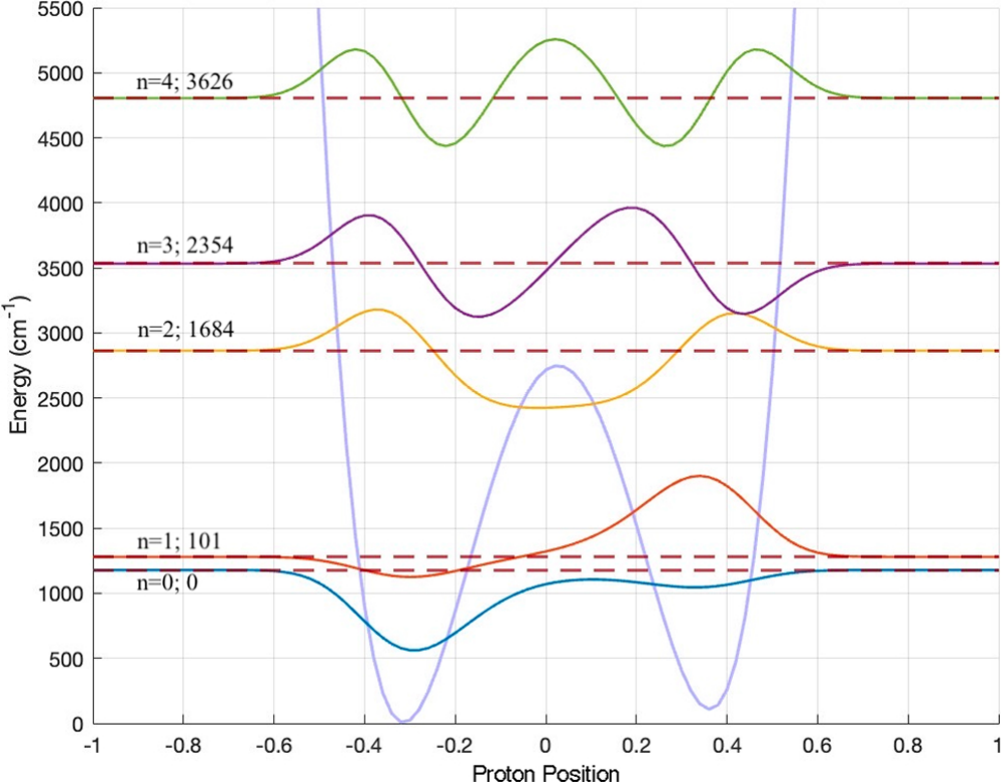
Potential energy path for the transfer of a proton from a nitrogen atom in a SB to an oxygen in a N_1_···H–O_1_ tautomer of **T2** (lavender); vibrational states and wave functions calculated for this potential—ground state (teal), first excited tunnelling state (red), second excited state (yellow), third excited state (violet), and fifth excited state (green). Relative energy of each vibrational state is in cm^–1^. Position of the hydrogen atom is measured from the midpoint of the N_1_···O_1_ separation, 1.375 Å from the nitrogen and the atom.

In the calculated energy path (the lavender color curve in Figure 4), the minimum energy of the neutral tautomer is only 112.8 cm^–1^ above the minimum energy of the ionic structure. Due to the constrained N···O distances, the potential energy barrier for proton transfer is much higher than the barriers present for the relaxed system. It is 2742 cm^–1^ above the minimum of the ionic structure.

The dynamic calculations indicate that proton transfer is feasible. The obtained wave functions are displayed in Figure 4. They are overlaid onto the calculated energy variation along the proton transfer path. In the ground state, there is some probability of finding protons in the neutral form. The first excited state is mostly in a neutral tautomeric form. It is 101 cm^–1^ above the ground state, a similar energy shift to the difference between the lowest energy local minimum and the global minimum. The second excited state, at 1684 cm^–1^, and the fourth excited state, at 2354 cm^–1^ above the ground state, are above the barrier. These energies are lower than the energies of stretching vibrations of N–H or O–H bonds, in a medium-strength hydrogen bond. When tunneling of both protons in a hydrogen bond/SB is considered, at least one more very low-energy state will be present.

These results indicate that in a low-polarity environment, tunneling between an SB and neutral tautomers of an Arg and acidic residue pair is feasible. Leu may be responsible for adjusting the polarity of the environment around an SB and optimizing the energy gap between ionic and neutral structures, when such low-energy states are needed. The presence of low-energy, thermally accessible states will increase entropy and specific heat. Fluctuations of inter- and intramolecular distances contribute further to increasing entropy. Finally, polarizability will be increased by the fluctuation of protonic charge, in addition to electronic charge. This will enhance the dispersion between Leu and the Arg-Acid pair. Deng and Cui found that electronic polarization is essential to the stability, hydration, and dynamics of buried charges in proteins.^43^

#### Importance of Leu near SBs

Analysis of SBs involving Arg in the PDB files by Donald et al. reveals that the fundamental geometry of SBs remains largely invariant for different solvent accessibility of protein surfaces.^12^ Thus, the behavior of SBs inside a protein is similar to that on the surface. For crystal structures, that behavior can be related to the fact that in small cavities the dielectric constant is small.^44,45^ In the packed environment of a crystal, there is no environment simulating liquid water. It is known that results obtained from NMR measurements in solutions do not always agree with the results of scattering experiments from crystals.^46^

Perl et al. reported that two AAs on the surface of the cold shock protein *Bc-Csp*, Arg3, and Leu66, are important for the protein’s thermostability.^47^ The temperature of the midpoint of thermal unfolding, *T*_m_, for this protein is 77°. The double mutation, R3E/L66E, to AAs present in the homologous mesophilic protein *Bs-CspB*, leads to the decrease of *T*_m_ by 32.3 °C. The reciprocal double mutation, E3R/E66L, in the mesophilic protein *Bs-CspB*, increased the *T*_m_, originally at 54 °C, by 20.6 °C. Although the destabilization of the mesophilic protein could be due to the repulsion of charged glutamate residues, the importance of Leu for the increase of stability was documented.^47^

Are thermophilic proteins more rigid or more flexible than their mesophilic homologues? Karshikoff et al. reviewed the role of fluctuations on protein thermostability.^48^ Increased mobility and fluctuations are important for psychrophilic enzymes in order to facilitate faster transport and reactions at lower temperatures. Increased flexibility was, however, found in thermophilic proteins, as well. Fitt and Heberle compared fluctuations in mesophilic and thermophilic homologues of α-amylase by monitoring hydrogen/deuterium exchange with FT-IR.^49^ They also used incoherent neutron scattering to monitor local equilibrium fluctuations in each of the homologues. Unexpectedly, they found a higher structural flexibility of the thermophilic α-amylase. This flexibility was already higher in the native, folded state, indicating a lower change of entropy or a lower change of *C*_*p*_, upon unfolding.^49^

SBs on protein surfaces contribute to the increased stability of thermophilic proteins, as supported by our calculations.^19,20,50^ Change of configurational entropy and specific heat, between folded and unfolded states, was demonstrated to be smaller in thermophilic enzymes relative to their mesophilic homologues.^19,48,49,51,52^ As observed by Fitt and Heberle, the flexibility of thermophilic proteins is already higher in the folded native state. We can attribute that to the increased density of low-energy, thermally accessible states due to hydrogen delocalization in SBs containing an Arg side chain with the proximity of a Leu side chain. Larger numbers of SBs on the surfaces of thermostable proteins contribute to the increased stability.

Kumar and Nussinov published several papers analyzing the factors contributing to the stability of thermophilic proteins.^20,53,54^ For our study, the most important is their finding is that psychrophilic and hyperthermophilic citrate synthases share larger sequence and structural similarities of SBs with each other than with the mesophilic homologue.^55^ An increased number of fluctuating SBs in psychrophilic enzymes increases their flexibility and allows them to work effectively at low temperatures.

In the NMR investigation of the isolated voltage-sensing domain (VSD) of the KvAP potassium channel, Shenkarev et al. detected μs–ms exchange processes in the interior of the S1–S4 helical bundle, around Asp62:Arg133 SB.^56^ They observed a single set of NMR signals for all VSD residues, indicating that the exchange process is fast on the chemical-shift time scale, and concluded that these fluctuations are of low amplitude. In the crystal structure of the VSD channel, Arg133 is 3.459 Å from Leu129. We postulate that the NMR observed exchange process corresponds to the proton at different locations. The electron shielding of neighboring atoms should oscillate with the proton position—negatively charged oxygen interacts with its surroundings in a different manner than the neutralized oxygen after accepting a proton.

The importance of the Leu residue for the increased stability of an SB and the whole protein can be deduced from the work by Sakaguchi et al.^57^ They found that a highly conserved SB between Arg and Asp side chains is very important for conferring structural stability to proteinase K subfamily enzymes, the thermophilic Aqualysin I, as well as the homologous psychrophilic serine proteinase. The disruption of this SB in Aqualysin I led not only to a significant reduction of the thermal stability but also to the disruption of the secondary structure. The SBs and their surroundings are nearly identical in the crystal structures of both proteins (see Figure 5). One of the Leu has one of the methyl groups directed toward the amino group of the Arg side chain. The second one has three carbon atoms over and below the SB. We suggest that protons in this SB are in a dynamic equilibrium between ionic and neutral forms. The increased fluctuations in the psychrophilic enzyme are important for increased mobility and transport at lower temperatures. For the thermophilic enzymes, the higher density of low-energy vibrational states, leading to increased specific heat and entropy, is important. The increased flexibility of the thermophilic enzymes, however, does not lead to their denaturation.^49^

**Figure 5:**
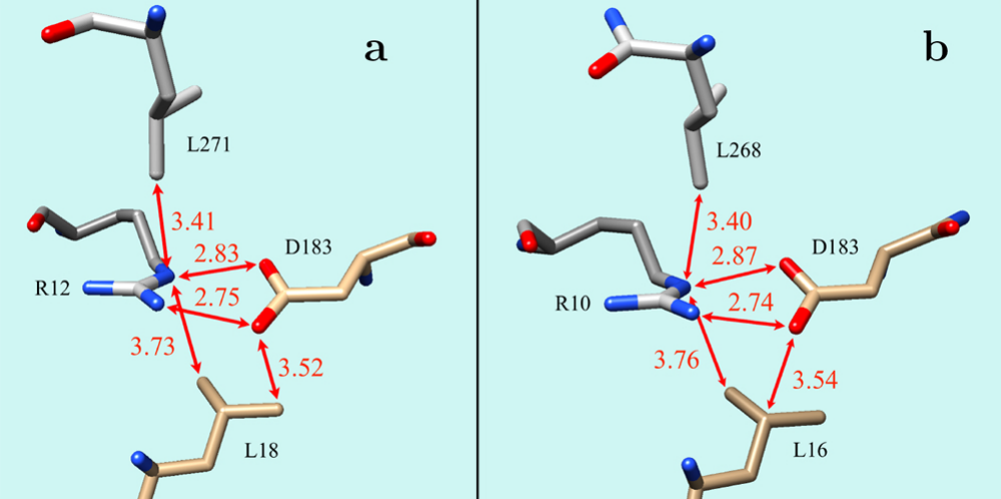
SB “sandwiched” between two Leu residues in (a) the thermophilic serine protease Aqualysin I [PDB ID 4DZT] and in (b) the cold adapted subtilisin-like serine proteinase [PDB ID 1SH7, chain A].

Another example of the increased stability of an SB interacting with Leu is provided in the NMR experiment of Mackenzie and Flemming-Hansen. The protection factor (PF) for the hydrogen exchange in the Arg residues of the T4 Lysozyme is about 600 times higher for Arg145, which forms a close contact with Leu7, than for Arg76, without Leu proximity.^58^ Both Args form monodentate SBs.

In reference to the two alternative locations of the Arg side chain in methionine adenosyltransferase 2A, shown in Figure 2, we propose that they correspond to different forms of the SB. When the methyl group of Leu42 is further away from the amino group (3.39 Å), the ionic form with shorter N···O distances dominates (Figure 2a), while the neutral forms with longer N···O distances (Figure 2b) dominate when the methyl group of Leu42 is closer to Arg292 (3.18 Å). The Leu42 approach to Arg292 seems to induce the shift of the amino proton to the oxygen of Glu39.

There are two tyrosine rings in the vicinity of this SB. We already observed the frequent presence of aromatic side chains in the proximity of Arg-Leu pairs when the Arg side chain did not form an SB.^8^ Further investigations are needed to fully explain the SB-methyl interactions and the influence of the surroundings, especially aromatic residues, on the dynamics of hydrogen bonds/sSBs between Arg side chains and acidic residues.

SBs on protein surfaces were indicated as an important factor to the enhanced stability of the thermophilic proteins.^18,20,50^ What is the behavior of the water of hydration around the SB-Leu complex on a protein surface? Will water form a protective shell around such SBs with fluctuating charges? A regular arrangement of hydrating water was not observed around the hydrophobic surfaces of proteins.^59^ Fluctuating charges of an SB may induce a fluctuating network of water surrounding the SB, which adds to its stability. Considering the large differences in water properties near 0 °C and near 100 °C,^55^ the dynamics of such hydration waters may be different for psychrophilic proteins and for thermophilic proteins. Pure water structure is still an enigma, and predicting its hydration effects on the entire protein’s surface with confidence is presently difficult.^59,60^

#### Second Protonation of Guanidine

While inspecting the PDB structures, we found that some of the observed N···C distances between the Arg and Leu side chains were shorter than the distances obtained in our calculations. In order to increase attraction between mGdnH^+^ and TMB, we doubly protonated mGdn and included the results in Paper I.^8^ Doubly protonated guanidine was observed in the NMR experiment of Olah and White,^61^ and recently, it was found in a crystal structure.^62^ In the doubly protonated dimer, the distances between hydrogen atoms decreased, and the energies of the complexes were lowered, relative to the energies of complexes with monoprotonated guanidine. After the second protonation of the mGdn in the TMB·mGdnH_2_^+^·Ac^–^ trimers, we observed a lowering of the energy as well.

Energies of a few optimized trimers with the doubly protonated mGdn are presented in Table 2. These structures are displayed in Figure S4. In each case, the initial structure was built by adding a proton to a puckered nitrogen atom with an elongated C–N bond close to the supporting methyl group(s) of TMB. The location of the extra protonated amino group, relative to the SB, affected the final structure and the energy.

**Table 2:** Energies of Structures with Doubly Protonated Guanidine in Close Contact with a Leu Side Chain, **E**, Calculated with the Counterpoise Correction^a^

	*E* (E_h_)	Δ*E*_*int*_ (kcal/mol)			3-fragments			
str.	3-fragments	3-fragments	TMB-SB @3-frag. geometry	TMB-SB 2-frag. optimized	TMB···mGdnH_2_^2+^ (Å)		mGdnH_2_^2+^···Ac^–^ (Å)	
bidentate					N···C	H···H	N_1_···O_1_	N_2_···O_2_
**X1**	671.973151	–30.76	–11.47	–11.47	3.366	2.002	2.885	2.712
**X2**	–671.965698	–31.71	–11.58	– 11.58	3.298	1.740	2.710	2.874
X3a	–671.965340	–31.17	–11.08	–11.08	3.285	1.883	2.710	2.872
**X3b**	–671.963800	–29.44	–10.98	–10.98	3.305	1.901	2.873	2.728
**X4**	–671.963230	–28.09	–10.38	–10.38	3.346	1.747	2.894	2.739
monodentate after proton transfer					N···C	H···H	N_1_···O	N_2_···O
**X5**	–672.032108	–26.65	–6.91	–6.91	3.463	2.147	2.873	2.906
**X6**	–672.032057	–26.84	– 6.89	– 6.89	3.532	2.084	2.853	2.905
**X7**	–672.031646	–26.39	–6.80	–6.80	3.497	2.189	2.871	2.840
**X8**	–672.031571	–26.69	–7.91	–7.90	3.522	2.617	2.878	2.858

a Interaction energy, Δ**E**_*int*_, for the trimer optimized with the three-body counterpoise correction; for the TMB interacting with the SB at the optimized geometry with the 3-fragments counterpoise correction; for the TMB interacting with the SB optimized with the two body counterpoise correction. Shortest distances N···C, H···H, N_1_···O_1_, and N_2_···O_2_ between monomers in the trimers.

For the optimization of **X1–X4** structures, protons were added to the amino groups of mGdnH^+^, away from the SB. During the optimization, such protons stay in the initial well, on the nitrogen with the elongated N–C bond. However, in each case, a proton in an SB is transferred to the acetate ion, while the bidentate arrangement remains. The structures with a proton shifted to the acetate have the lowest energy. The structure with an ionic Ac^–^ becomes a transition state. In Figure S7a, an example of a transition between structures with a neutral HAc is displayed. The relative energy of the transition state is 3358.2 cm^–1^ above the minimum energy of the **X3a** structure. It remains to be determined if the transition between two tautomeric structures with a neutral HA side chain is feasible. Even more important is whether a trimer with the doubly protonated Arg side chain is stable.

In the **X5–X7** structures, a proton was added to the amino group involved in an SB. In this case, a proton from the NH_3_^+^ group is immediately transferred to the acetate ion, and the mGdnH+ is converted to its lowest energy, planar configuration. The repelled hydroxyl group of HAc rotates away from the guanidinium ion. The amino groups of the planar mGdnH^+^ form a monodentate hydrogen bond with the carbonyl of the HA. The initial and the optimized final **X5** structures are displayed in Figure S7b. A few snapshots from the optimizations are included in the figure.

Could a proton on an amino group away from the SB be transferred to an amino group in an HB? Is the interaction of the NH_3_^+^ group with the Leu side chain sufficiently strong to prevent the transfer of the extra proton to a neighboring amino group? The answer is important for the determination of the stability of the doubly protonated complex. Guanidinium protons of Arg are generally labile and able to exchange with the bulk solvent.^58^ We believe that the extra proton will be able to transfer to a neighboring amino group; however, in order to get a definite answer, more investigations are needed.

In the trimers with doubly protonated Arg side chains, our calculations yield very close distances between hydrogen atoms of the Arg and Leu side chains, with the longest distance at 2.002 Å. In the dimers, the H···H distances were even shorter, in the range 1.440–1.805 Å.^8^ Are the hydrogen atoms of the Arg and Leu side chains exchanged during the interaction? Scrambling of dihydrogen-bonded protons was observed in dihydrogen-bonded complexes involving transition metals.^11^ Methyl group labeling in the NMR experiments,^63^ or an analysis of the protein fragments in GC/MS, may answer the questions regarding whether or not the exchange takes place.

The energy of the protonated trimers (with the doubly protonated Arg side chain) is lower than the energy of the neutral trimers (with the monoprotonated Arg side chain). The energy of the trimers, after proton transfer and mGdnH+ returning to its lowest energy, the planar structure, is even lower. These results indicate that the second protonation of the Arg side chain may be a component of the proton transfer path. After the formation of a transient SB, the translocation of a proton to an acidic residue occurs. The remaining hydrogen bond is much weaker than an SB, and the separation of a neutral, protonated acidic residue from the Arg side chain is easier.

Such transfer may occur in ion channels in membranes, where SBs are embedded between aliphatic and aromatic side chains.^64^ While in this work we focused on Leu in close contact pairs with Arg, methyl groups of other branched aliphatic residues, as well as threonine, or methionine, may be involved in similar interactions with Arg side chains. Several examples of the guanidine moiety of Arg, “sandwiched” between various nonpolar side chains, are presented in Figures 1, 6, 7, and S7 of ref 64.

## Summary and Conclusions

We inspected high-resolution PDB files that contained triplets of Leu, Arg, and acidic residue side chains in a close approach. We also performed DFT calculations of the interaction energy between models of the three side chains: TMB, mGdnH^+^, and Ac^–^. The wB97X-D density functional and the aug-cc-pVTZ basis set were used in the calculations. The interaction energy of Leu and an SB was obtained from the calculations using the structure optimized with the three-body counterpoise corrections. The trimers were optimized in a vacuum as well as in chloroform by using the PCM method. In addition to the structures with an SB, we optimized tautomeric structures with neutral mGdn and HAc.

Calculations of SB structures, involving Arg, yield too short distances between monomers in SBs. Embedding an SB in a higher dielectric constant environment increases N···O separations. However, even in a water environment, this separation is too small in order to account for the longer distances observed in the PDB crystal structures of proteins. In addition to the ionic structures, we optimized the structures of two neutral tautomers of each ionic conformer.

The N···O distances in the neutral tautomers are much longer than those in the ionic conformers. We suggested that a dynamic equilibrium between three conformers in an SB can account for longer N···O separations in an SB, explaining the longer distances observed in protein crystal structures. The trial 1-D quantum mechanical calculation of the dynamics indicated that a transition between three conformers is feasible in a low dielectric constant environment. In the low dielectric constant environment, the minima of the ionic conformer and its neutral tautomers have similar energy, although their intermonomer separation differs significantly. Tunneling between these conformers yields large amplitude fluctuations in an SB, as well as in the backbone and surrounding AAs that respond to the fluctuating charge in the SB.

The low-energy tunneling states increase the density of states accessible at ambient temperature and, thus, increase entropy in a native state. Thermophilic proteins are known to have a smaller increase in entropy upon a transition from a native to an unfolded state. The presence of a large number of fluctuating SBs in a protein may explain its increased thermophilicity. An increased number of fluctuating SBs also helps psychrophilic proteins to retain their activities at low temperatures. We propose that Leu residues, interacting with the amino groups of SBs, control the ratio of the ionic-neutral tautomers in dynamic equilibrium. They also provide a low dielectric constant environment of SBs. We present several examples of SBs, with a Leu residue in a close approach, that contribute to the stability of proteins.

The decreased distance between the carbon atoms of Leu and the nitrogen atoms of Arg can be achieved by the second protonation of the guanidine moiety of Arg. We propose that the doubly protonated trimers of Leu, Arg, and an acidic residue participate in proton transfer, especially in membrane proteins.

## Conclusions

- The distances in the SBs of the optimized structures in vacuum are quite short, on the order of 2.6 Å, much smaller than the distances observed in the crystal structures.
- The N···O distances in ionic forms increase in the calculations with the increasing dielectric constant, however, not sufficiently to account for the distances observed in the crystals.
- The N···O distances in the optimized neutral tautomers are much longer than in the ionic conformers. In a low polarity environment, the energies of the ionic and the neutral tautomers are close.
- In a low-polarity environment, an SB is in a dynamic equilibrium between an ionic form (SB) and two neutral tautomers, forming a hydrogen bond. The neutral tautomers with longer N···O distances contribute to the increase of the average N···O distances, bringing them to the range of values observed in crystals.
- We propose that Leu close to an Arg side chain controls the displacement of a proton from an amino group to a carbonyl oxygen, the relative energies of ionic and neutral tautomers, and the formation of thermally accessible, low energy tunneling states.
- Fluctuations in SBs, and their environments, increase the entropy and the specific heat. An increased number of fluctuating SBs in thermophilic proteins increases their entropy and their thermal stability, while in psychrophilic enzymes, facilitates faster transport and reactions.
- The decreased distance between the carbon atoms of Leu and the nitrogen atoms of Arg can be achieved by the second protonation of the guanidine moiety of Arg. The doubly protonated trimers of Leu, Arg, and an acidic residue may participate in a proton transfer, and lead to a translocation of a proton to an acidic residue.

Our conclusion that a neutral structure of the Arg side chain is in a dynamic equilibrium with its protonated form in a SB does not invalidate the conclusion of Harms et al. that “Arginine residues at internal positions in a protein are always charged”.^6^ In paper I, we showed that interaction between Leu and the monoprotonated Arg side chains is stabilizing and it is stronger than the interaction between two neutral species. In the hydrophobic interior of a protein Arg side chain (without a SB) will be protonated.^8^ With the doubly protonated Arg, the interaction with Leu is even stronger, in the range of interactions of Arg with aromatic residues.

## Supplementary Material



## Notes

Additional details of the 1D quantum mechanical calculation of tunneling between the ionic and the neutral tautomeric structures; examples of close contact triplets from PDB; structures of optimized TMB·mGdnH^+^·Ac^–^ trimers; structures of **T2**, **T5**, and **T6** in vacuum and in chloroform; **T2** structures and energies at different separation of SB monomers; N–H bond lengths in **T2** at different separation of SB monomers; optimized structures of the trimers with doubly protonated mGdn; evolution of the trimer structures after the second protonation of mGdn; Leu·Arg·Acidic residue triplets in a close contact from PDB; energies of neutral TMB·mGdn·HAc tautomers of **T2**, **T5**, and **T6**; and comparison of the energies and structures of mGdnH^+^·Ac^–^ and mGdn·Ac pairs calculated with the ωB97X-D density functional and the MP2 method (PDF)
