# Supplementary material for: Arginine: II. Interactions of Its Salt Bridges with Branched Aliphatic Side Chains
Source: J Phys Chem B. 2025 Jul 9;129(29):7430–41. doi: 10.1021/acs.jpcb.5c02172 (PMC12302201; doi:10.1021/acs.jpcb.5c02172)
Supplement: Supplementary file 1 [file jp5c02172_si_001.pdf]

Supporting Information

Arginine: II. Interactions of its Salt Bridges with Branched Aliphatic Side Chains

Christopher M. Ng, Vivian Kui, Katherine Y. Han, Eric R. Kempson, Margaret Mandziuk

Department of Chemistry, New York University, New York, USA

1-D quantum mechanical calculations of the dynamics

Allowing proton transfer without N−O distance constraint leads to a large elongation of the intermolecular distance for which potential is much softer. That is why we held the N−O distances constant at 2.75 Å. The energy path was calculated for structure **T2**, moving the proton from N_1 of the ionic structure (N_1−H$\cdots$$O_1$), to O_1 of the neutral structure ($N_1\cdots$H−O_1). The constrained optimization was performed at the ωB97X-D/aug-cc-pVTZ level. The second proton position was not constrained. It varied between 1.02 to 1.06 Å, close to its nitrogen atom.

The table below contains energy values as a function of the N$\cdots$H distance. These values serve as the potential, V, for the motion of hydrogen atom. In the second column, the coordinate value used in the calculations is given. The center of the coordinate system is placed in the center of the N$\cdots$O distance, 1.375 Å from the nitrogen atom.

N$\cdots$H	x (Å)	V (cm^{-1})
1.0	−0.375	431.05
1.0604	−0.3146	0.00
1.1	−0.275	135.64
1.2	−0.175	1174.19
1.3	−0.075	2278.80
1.4	0.025	2741.90
1.5	0.125	2318.31
1.6	0.225	1230.59
1.7	0.325	214.87
1.7335	0.3585	112.81
1.8	0.425	554.39

These values were fitted to a quartic polynomial $V(x) = a_0 + a_1x + a_2x^2 + a_3x^3 + x_4x^4$. With the coefficients $a_0 = 2716$, $a_1 = 2488$, $a_2 = -4.635 \times 10^4$, $a_3 = -2.056 \times 10^4$, and $a_4 = 2.062 \times 10^5$ RMSE is 8.063 cm^{-1}. Local minimum for the neutral structure is 112.81 cm^{-1} above the global minimum of the salt bridge and the barrier is 2741.90 cm^{-1} above the global minimum.

The 1-D Hamiltonian,

$$H(x) = -\frac{\hbar^2}{2m_p}\frac{d^2}{dx^2} + V(x),$$

was used, with m_p mass of a proton (1a.u.).

The dynamics was calculated using the in-house created Python code based on the DVR formulation of the solutions by Bačić and Light [Annu. Rev. Phys. Chem. *40* (1989) 469-498]. Gaussian quadrature points of scaled Hermite functions were used as the basis set. The lowest five states, shown in the figure below are converged to more than eight figures. The energy of the vibrational states, relative to the ground state, are shown also in the figure below.

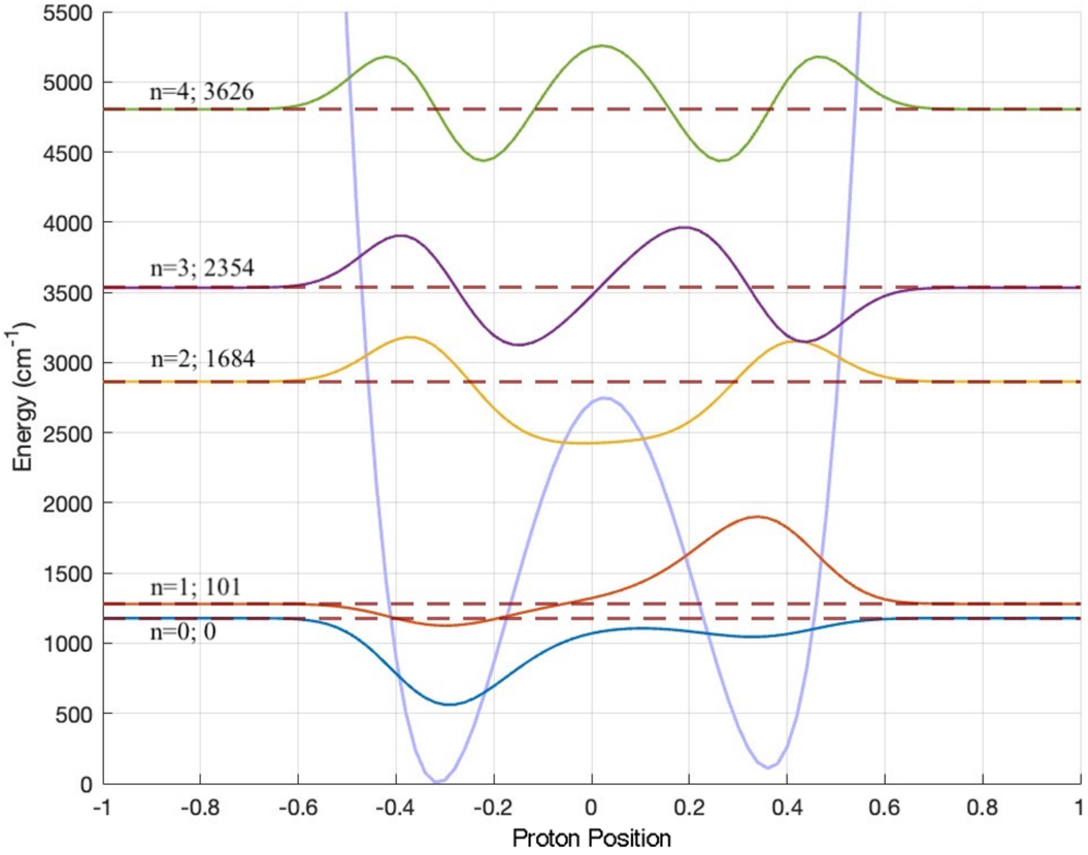

The ground and the first excited states have the character of tunneling states in a slightly asymmetric potential. The ground state is localized mostly above the well of the ionic form, with small contribution from the neutral tautomer, while the first excited state is mostly localized above the neutral form with a small contribution of the ionic form. These states are separated by 101 cm^{-1}, close to the value of the energy difference between the wells, 113 cm^{-1}.

The code for the calculations of the vibrational states is available in the GitHub repository:
https://github.com/cn2673/1D-Quantum-Mechanical-Dynamics-Calculations-Using-DVR-with-Hermite-Functions-in-Python

Figure S1. Examples of salt bridge structures with Leu residue approaching closely guanidinium moiety of Arg. (a) PDB ID 7B1S; (b) PDB ID 4UA6; (c) PDB ID 1BYI; (d) PDB ID 4TXR; (e) PDB ID 1O7J; (f) PDB ID 2JFR.

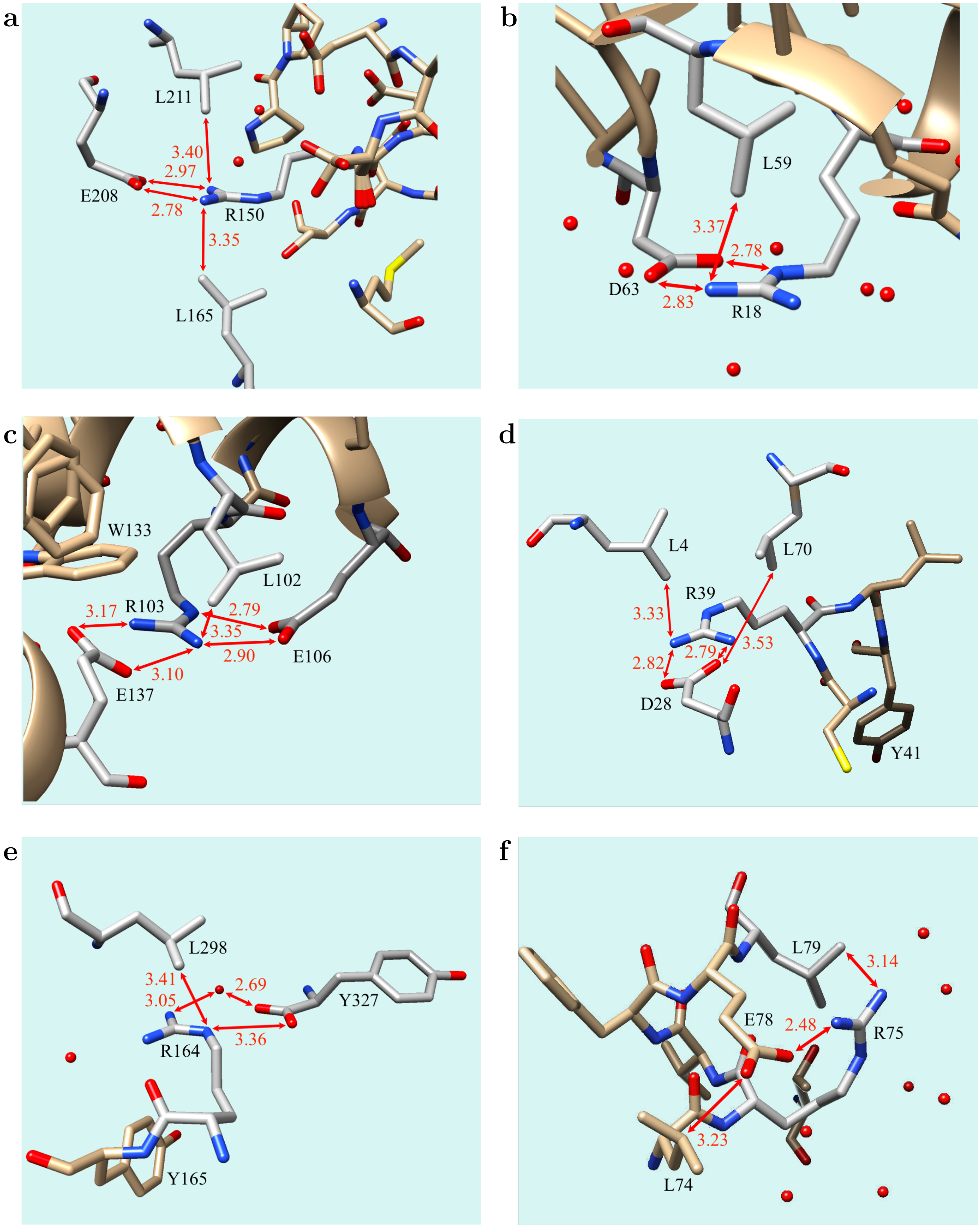

Figure S2. Structures of the trimers TMB·$GdnH^{+}$·Ac^{-} optimized in this work. Corresponding energies are shown in Table 1 in the main body of the paper. The shortest distance between hydrogen atoms of TMB and $GdnH^{+}$, the shortest distance between nitrogen atom of the Arg side chain and the Leu side chain models, as well as the distances between nitrogen atoms and oxygen atoms in salt bridges are displayed. Also shown is the figure with the electrostatic potential (EPS) mapped onto the electron density surface, drawn at $0.007\,e/bohr^3$. This value of electron density was selected to facilitate comparison with the EPS shown for dimers between $mGdnH^{+}$ and TMB, presented in Paper I [Ng *et al.* https://doi.org/10.1021/acs.jpcb.5c02168]. The range of the ESP is between -0.325 and 0.325 a.u. of ESP.

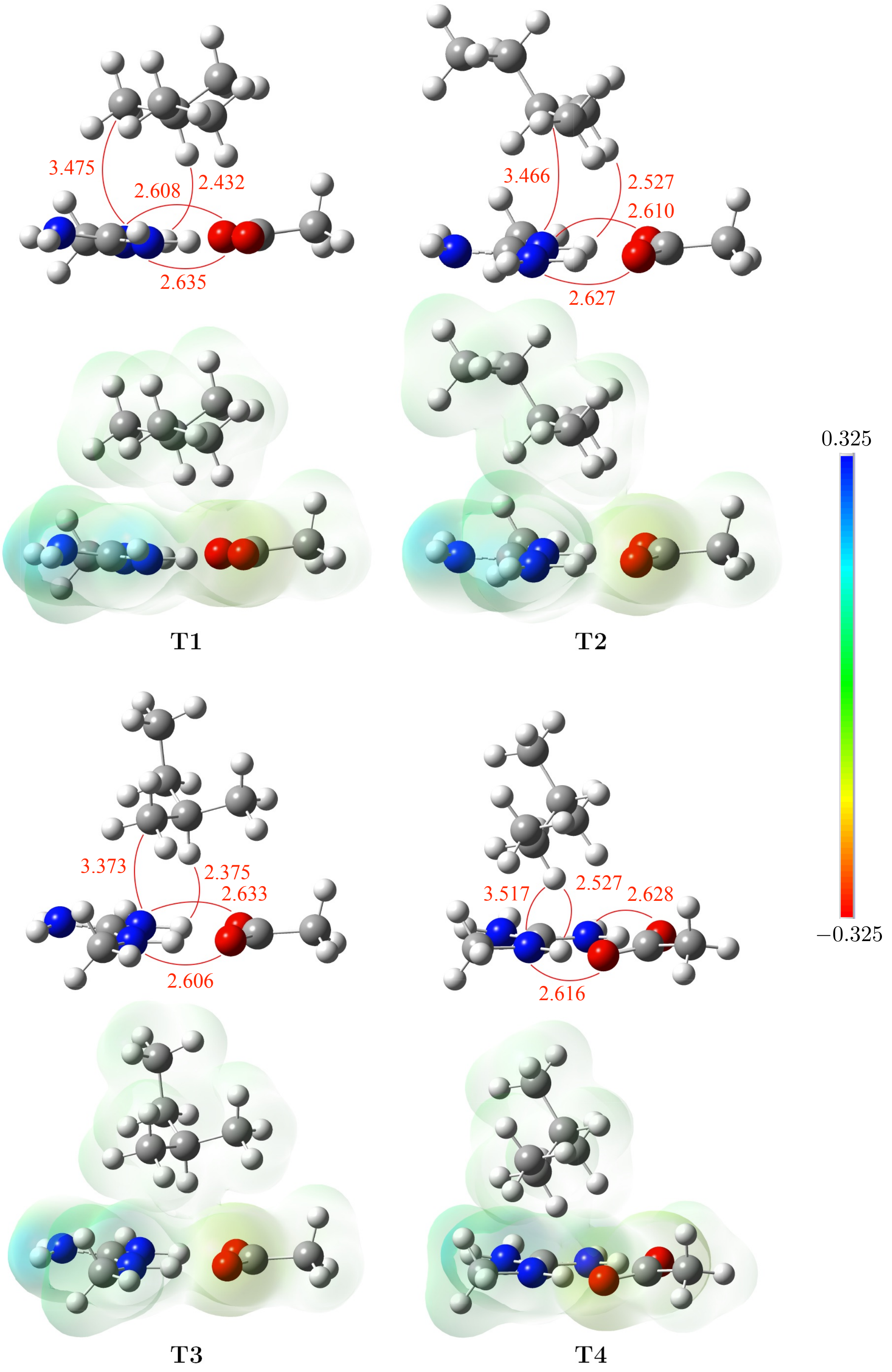

Figure S2 (continuation)

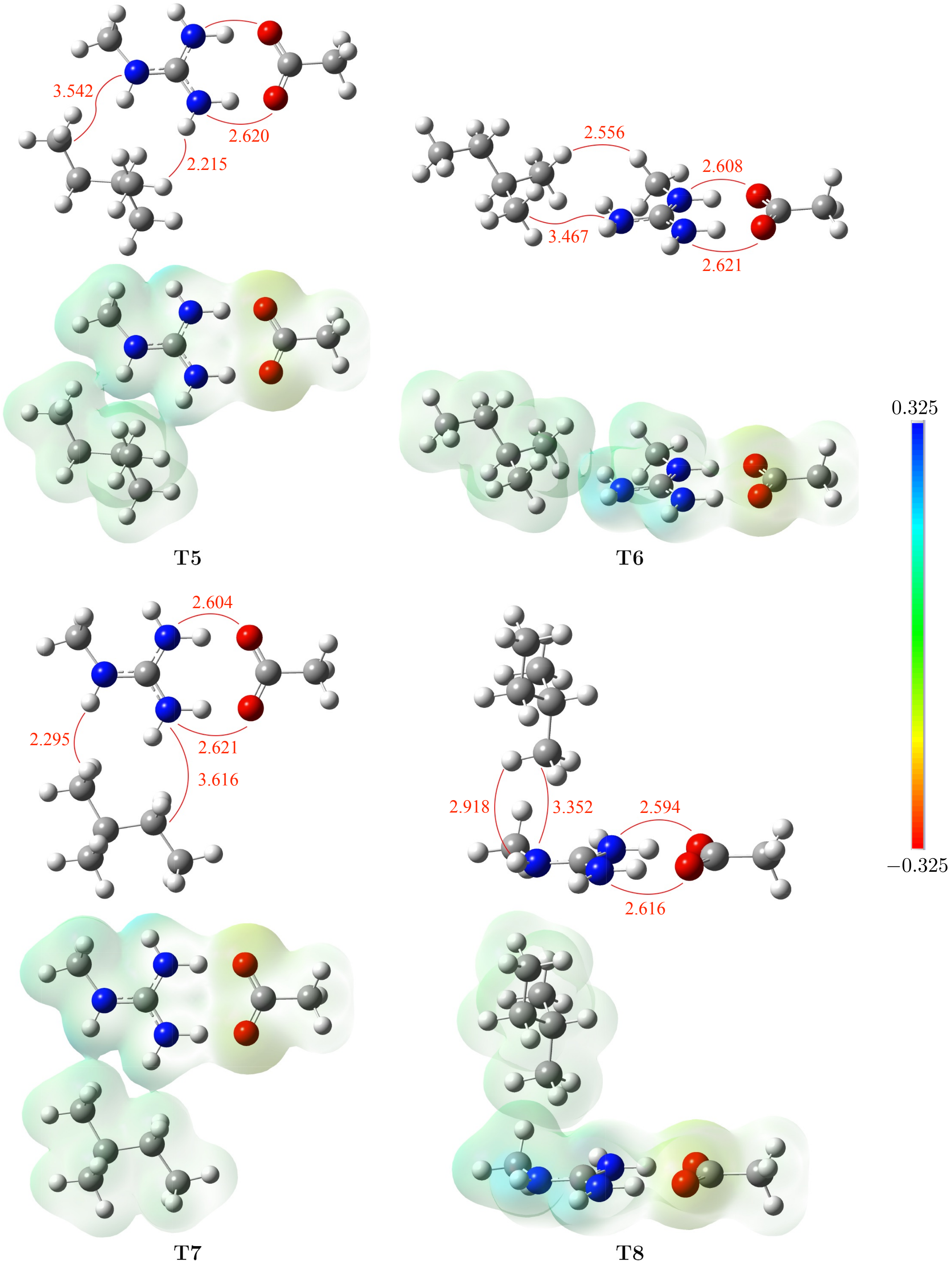

Figure S3. Optimized structures of the ionic and the neutral tautomers of **T2**, **T5**, and **T6** in a vacuum and in chloroform. Energies of these structures are not counterpoise corrected. Energy of each neutral tautomer, relative to the ionic form, is shown in bold (cm^{-1}). Small numbers in parentheses show the values calculated for the mGdnH$^+$·Ac$^-$ without TMB (from Table S2). Numbering of nitrogen and oxygen atoms is the same as in Fig. 3 of the main text.

	Salt-Bridge	$N_1 \cdots H{-}O_1$	$N_2 \cdots H{-}O_2$
		**T2**	
vacuum	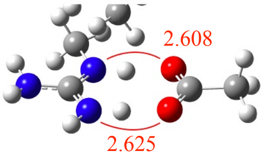 **0.0** −671.6285385	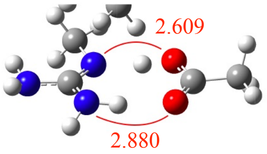 **324.1** (−58.5)	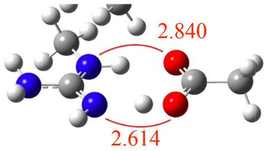 **183.7** (−74.9)
chloroform	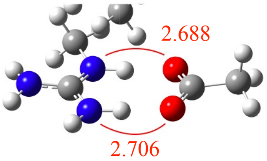 **0.0** −671.6447467	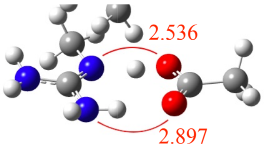 **1902.1** (1839.4)	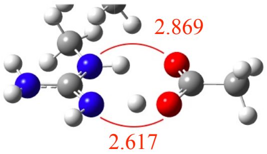 **1835.2** (1734.4)
		**T5**	
vacuum	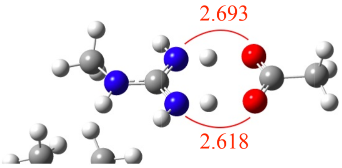 **0.0** −671.6253522	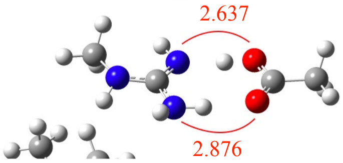 **−419.3** (−592.0)	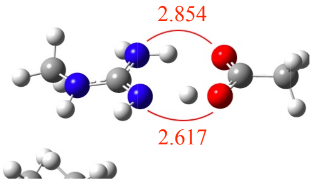 **11.9** (−133.0)
chloroform	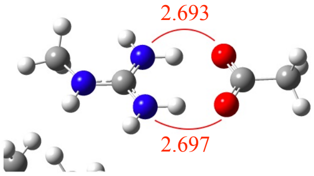 **0.0** −671.6417067	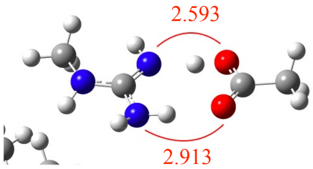 **1388.5** (1409.2)	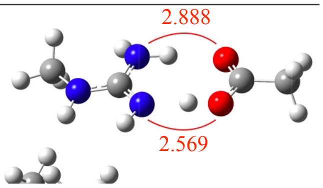 **1665.9** (1755.7)
		**T6**	
vacuum	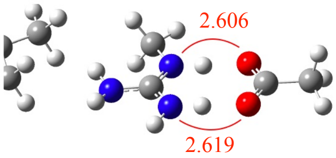 **0.0** −671.6240701	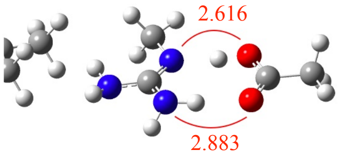 **74.6** (−58.5)	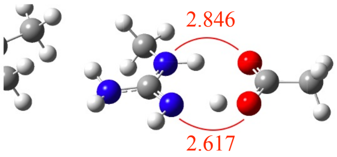 **41.4** (−74.9)
chloroform	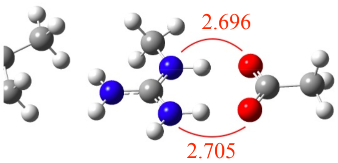 **0.0** −671.6405618	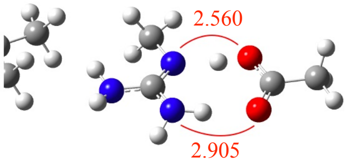 **1846.7** (1839.4)	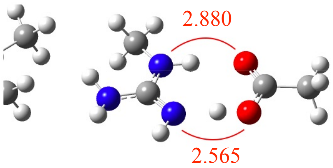 **1748.0** (1734.4)

Figure S4. The structures and the relative energies (cm^{-1}) of **T2** with the constrained distance (C···C) between central carbon atoms of $mGdnH^+$ and Ac^- (or their neutral tautomers) in vacuum (a) and in chloroform (b). Numbering of nitrogen and oxygen atoms is the same as in Fig. 3 of the main text.

a. Vacuum

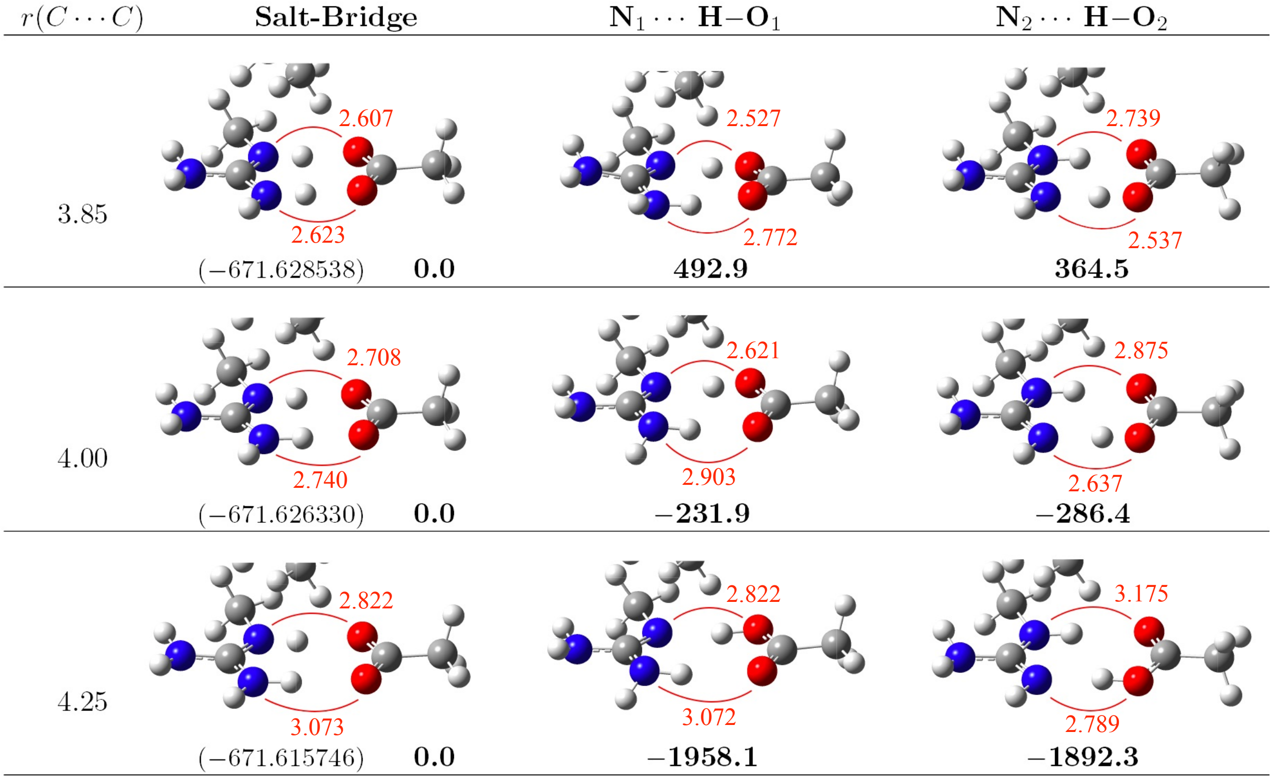

b. Chloroform

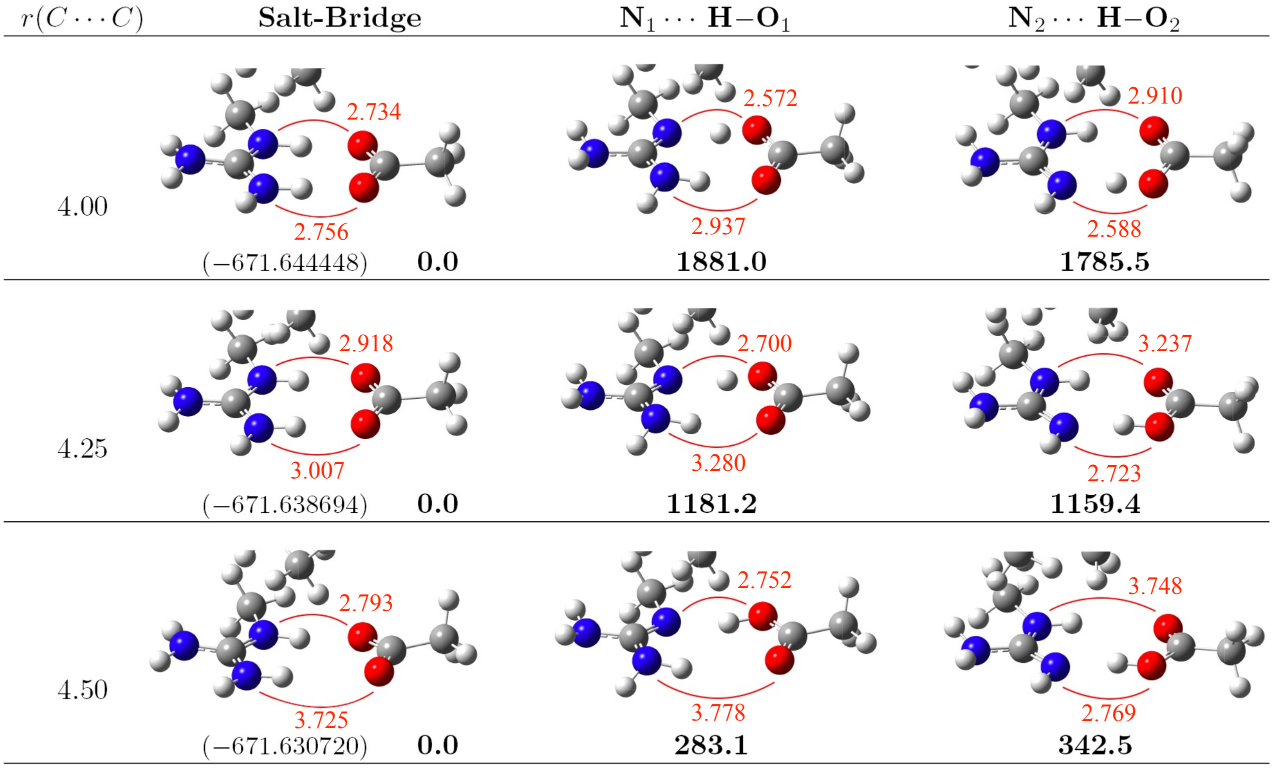

Figure S5. N−H bond lengths in salt bridges of the **T2** structure, optimized for a few fixed distances between central carbon atoms of $mGdnH^{+}$ and Ac^{-} moieties. With the increasing distance between monomers, N−H bond lengths in bidentate structures decrease in vacuum (a), as well as in chloroform (b). At the C···C separation of 4.5 Å, the SB opens up towards a monodentate structure.

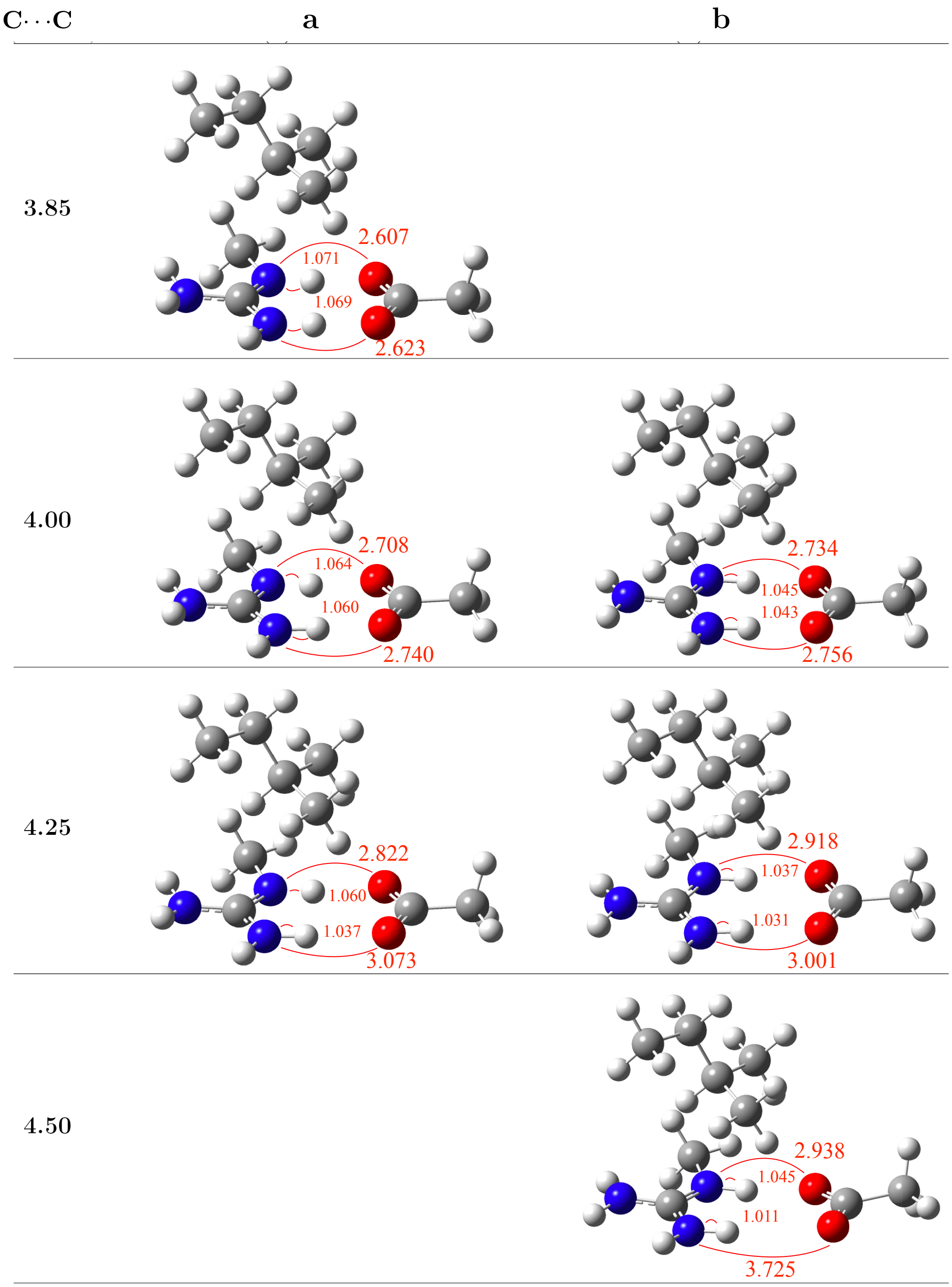

Fig. S6. Optimized structures of some salt bridges with the doubly protonated methylgaunidine, mGdnH_2^{2+}, supported by Leu side chain. Structures **X1**– **X4** are protonated on an amino group away from the salt bridge, while structures **X5**– **X8** were initially protonated on an amino group that participated in a salt bridge.

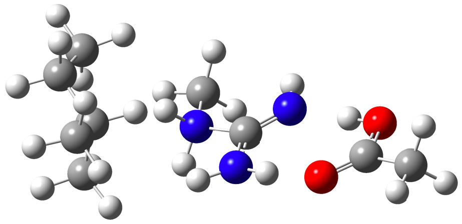

X1

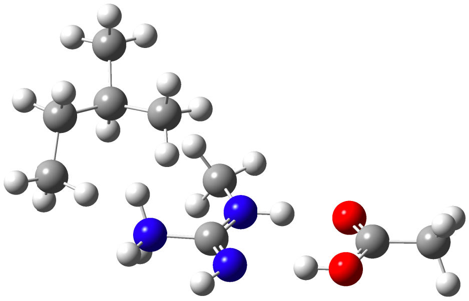

X2

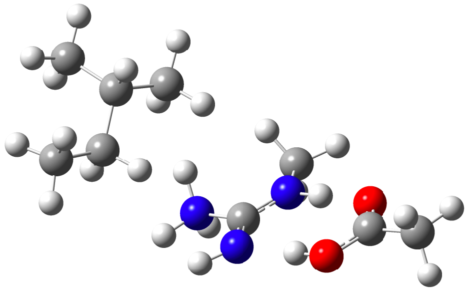

X3a

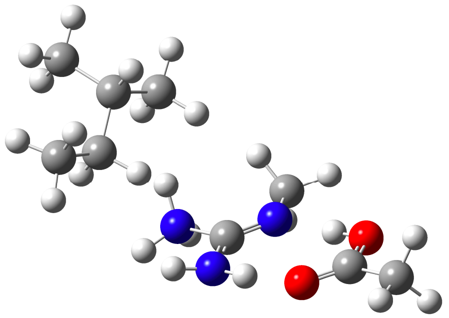

X3b

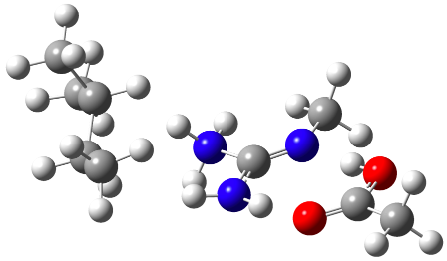

X4

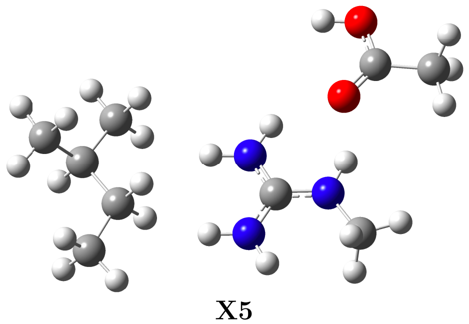

X5

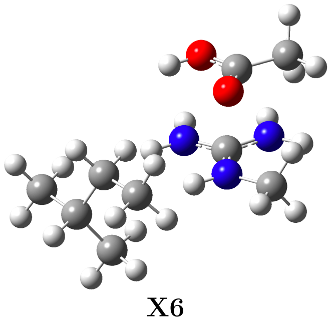

X6

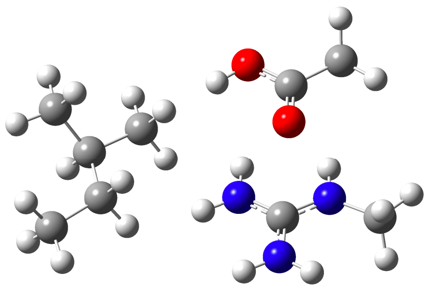

X7

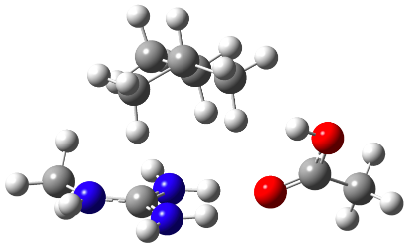

X8

Figure S7. Doubly protonated methyl guanidine. (a) Protonation of the amino group away from the salt bridge: structure with the doubly protonated mGdn and Ac^{-} is the transition state; extra proton stays on the same amino group during the optimization, however, a proton in the salt bridge is transferred to the acetate. (b) Protonation of the amino group participating in the salt bridge. Proton from the protonated group is immediately transferred to the acetate. Three panels below show snapshots from of the optimization. Guanidinium moiety becomes planar, O−H group of HAc rotates away from the amino group and the salt bridge becomes monodentate.

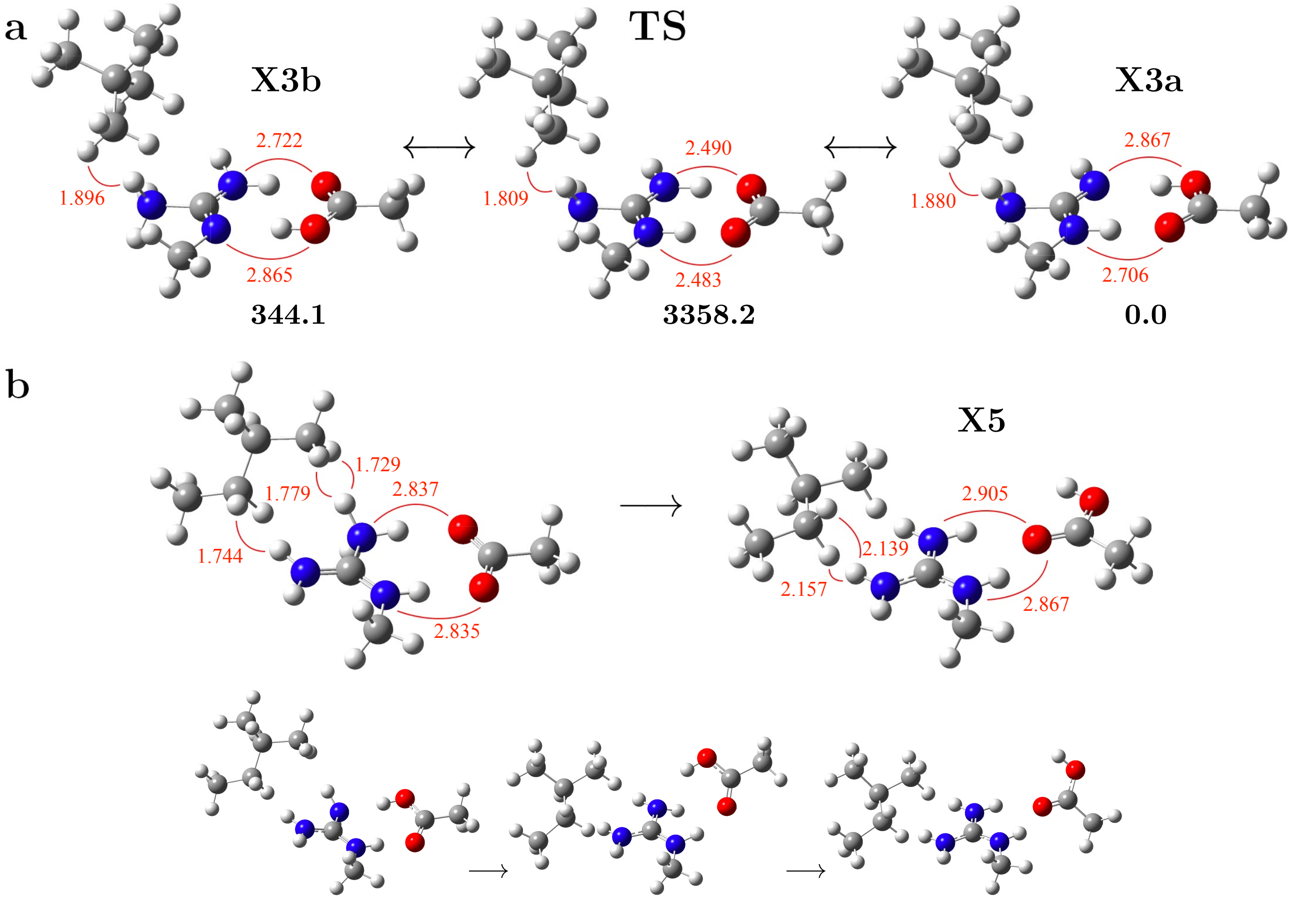

Table S1: The list of salt bridges with any of the Leu carbon atom approaching any of the Arg nitrogen atom to less than 3.5 Å. The closes distance between N of the Arg and O of the acidic residue in the selected salt bridge pairs is less than 3.5 Å. For Arg and Leu, the chain designation, residue number, and the ID of the atom making the closest contact, are given. In parentheses, the alternative location and its occupancy are given, if needed. In column 5, the acidic residue forming a salt bridge is listed, and in column 6 the shortest distance in the salt bridge is included. The last column lists the second closest distance in a given salt bridge. The number without parentheses indicates that the SB is a bidentate type. In a monodentate SB, the number in curly brackets indicates the distance from the N atom to the second O atom in the SB, while the number in square brackets indicates the distance between the O atom and the second N atom in the SB.

$(N-C)_{min}$ (Å)	PDB ID	residues			$(N-O)_{min}$ (Å)	second bidentate
		Arg	Leu	Acidic Residue		
2.76	2XFR	A:R34:NH1(B;0.50)	A:L76:CD1(B;0.50)	A:D31	2.67	
2.79	1O7J	C:R164:NH2	C:L298:CD2(B;0.27)	C:Tyr327(OXT)	2.83	(3.37)
3.13	5CKL	A:R158:NH2(A;0.52)	A:L155:CD1	A:D154	2.91	
3.14	2JFR	A:R75:NH2(A;0.63)	A:L79:CD1	A:E78	2.48	
3.18	7RWG	A:R292:NH1(B;0.36)	A:L42:CD2	A:D39	3.01	3.05
3.19	4UA6	A:R184:NH2	A:L59:CD1(B;0.59)	A:D63	2.78	2.83
3.24	2JFR	A:R40:NE(A;0.56)	A:L80:CD2	A:E83(A;0.80)	2.82	
3.24	7B1S	A:R243:NE(B;0.50)	A:L563:CD2	A:D560	2.79	[3.38]
3.25	2WFI	A:R170:NH1(A;0.79)	A:L172:CD2	A:E:168	2.94	
3.27	1NKI	A:R117:NH1	A:L120:CD1	A:E31<br>A:E41	2.85<br>2.87	3.44
3.28	7B1S	D:R243:NE	D:L563:CD2	D:D560	2.80	[3.39]
3.29	5DGJ	A:R89:NE(B;0.41)	A:L95:CD1	A:D138(B;0.53)	3.07	[3.22]
3.29	7ADR	F:R94:NE(B;0.50)	F:L6:CD2	F:D7	2.82	(3.27)
3.30	1JFB	A:R202:NH2	A:L198:CD1(A;0.50)	A:D209	2.99	
3.31	1MUW	A:R386:NE(B;0.44)	A:L377:CD2	A:D380<br>A:E324(A;0.60)	2.85<br>2.59	
3.31	1MUW	A:R386:NE(B;0.44)	A:L377:CD2	A:D380<br>A:E324(B;0.40)	2.85<br>3.11	
3.33	1O7J	A:R164:NH2	A:L298:CD1(A;0.64)	A:Tyr327(OXT)	2.82	(3.36)
3.33	4TXR	A:R39:NH2	A:L4:CD1	A:D28	2.79	2.82
3.35	1BYI	A:R103:NH2	A:L102:CD2	A:E106<br>A:E137	2.79<br>3.10	2.90<br>[3.11]
3.35	5GJI	A:R340:NH2	A:L380:CD2(B;0.37)	B:D190	3.27	
3.35	7B1S	F:R150:NH2	F:L165:CD1	F:E208	2.78	
3.35	3EO6	A:R27:NE(B;0.64)	B:L39:CD2	A:D100	2.90	[3.08]
3.36	3EO6	B:R27:NE	A:L39:CD2	A:D100	2.77	[3.07]
3.38	3EO6	A:R27:NE(A;0.36)	B:L39:CD2	B:D100 (+ -SO_3)	2.60 (2.94)	[3.05]
3.39	1O7J	C:R164:NH2	C:L298:CD1(A;0.73)	C:Tyr327(OXT)	2.83	(3.37)
3.39	7RWG	A:R292:NH1(A;0.65)	A:L42:CD2	A:D39	2.77	2.79

Table S1, continuation

$(N-C)_{min}$ (Å)	PDB ID	residues Arg	Leu	Acidic Residue	$(N-O)_{min}$ (Å)	second bidentate
3.40	7B1S	F:R150:NH1	F:L211:CD1	F:E208	2.78	2.97
3.40	7P24	A:R431:NE	A:L434:CD2	A:D396	2.76	2.82
3.40	1GKM	A:R355:NH1	A:351:CG	A:E441	2.67	2.84
3.41	1O7J	A:R164:NE	A:L298:CD1(B;0.36)	A:Tyr327(OXT)	2.82	(3.36)
3.41	1O7J	D:R164:NH2	D:L298:CD1	D:Tyr327(OXT)	2.83	(3.39)
3.42	1O7J	B:R164:NH2	B:L298:CD1	B:Tyr327(OXT)	2.81	(3.36)
3.42	1TT8	A:R51:NE	A:L75:CD2	A:E59	2.90	[2.97]
3.42	4TXR	A:R146:NH2(B;0.74)	C:L167:CD1	C:E164(B;0.33)	2.77	
3.42	5HB7	A:R160:NH1	A:L191:CD2	A:E195,<br>A:E214	2.85<br>3.12	3.14
3.43	2CNQ	A:R17:NH2	A:L14:CD2	A:D22	2.95	(3.25)
3.43	4G9S	A:R54:NH1(A;0.57)	A:L51:CD2	A:E110	3.25	[3.49]
3.44	1MJ5	A:R202:NH2	A:L164:CD1	A:E199<br>A:E160	2.80<br>2.93	2.82
3.44	5U3A	A:R124:NE	A:L166:CD2	A:D138	2.79	2.90
3.45	7B1S	C:R150:NH2	C:L165:CD1	C:E208(A;0.50)<br>C:E208(B;0.50)	2.97<br>2.99	 3.35
3.46	4EA9	A:R87:NE	A:L93:CD1	A:E111<br>A:D129	2.51<br>3.09	 [3.39]
3.46	5D66	A:R157:NH1(A;0.80)	A:L196:CD1	A:E154(A;0.70)	2.79	2.84
3.46	5D66	A:R157:NH1(A;0.80)	A:L196:CD1	A:E154(B;0.30)	2.96	
3.46	5D66	A:R219:NH1	A:L215:CD2	A:E227	2.77	[3.48]
3.46	5D66	B:R157:NH1(B;0.20)	B:L196:CD2	B:E154(A;0.70)	2.92	
3.46	5D66	B:R157:NH1(B;0.20)	B:L196:CD2	B:E154(B;0.30)	2.83	3.00
3.47	4G9S	A:R176:NH2	A:L37:CD1	A:D172	2.80	
3.47	4U9H	L:R38:NE	L:L364:CD1	L:E40<br>L:D28	2.80<br>2.87	[3.44]
3.47	5U3A	A:R85:NH2	A:L26:CD2	A:E29	2.87	2.88
3.48	4Y9W	A:R121:NH1	A:L182:CD1	A:E316	3.46	
3.49	4GA2	A:R3:NE	A:L35:CD2	A:E38	2.71	[3.09]

Table S2. Energies, E, of the neutral tautomers (TMB·mGdn·HAc trimers) of the **T2**, **T5**, and **T6** structures, optimized in this work with the counterpoise correction for three fragments. Interaction energy, ΔE_{int}, was obtained in calculations with the counterpoise correction for three fragments. It was also calculated for two fragments, TMB and the SB, in the geometry optimized for three fragments.

structure	E (E_h)	ΔE_{int} (kcal/mol)		N$\cdots$C	H$\cdots$H	$N_1\cdots O_1$	$N_2\cdots O_2$
		3-fragments	2-fragments @ 3-frag. geom.	(Å)			
**T2** ($N_1\cdots H{-}O_1$)	-671.626350	−28.72	−5.15	3.474	2.653	2.609	2.880
**T2** ($N_2{-}H\cdots O_2$)	-671.626638	−30.99	−5.35	3.493	2.552	2.840	2.614
**T5** ($N_1\cdots H{-}O_1$)	-671.626403	−27.59	−4.38	3.574	2.213	2.641	2.883
**T5** ($N_2{-}H\cdots O_2$)	-671.624407	−29.37	−4.43	3.635	2.216	2.861	2.621
**T6** ($N_1\cdots H{-}O_1$)	-671.622948	−26.44	−2.92	3.485	2.313	2.621	2.891
**T6** ($N_2{-}H\cdots O_2$)	-671.623097	−28.45	−2.97	3..485	2.284	2.852	2.621

Table S3. Comparison of energies of hydrogen bonded/SB pairs of mGdnH+ and TMB, optimized with the ωB97xd functional as well as with the MP2 method. The aug-cc-pVTZ basis set was used in each case. Calculations were performed in vacuum and in chloroform (clfm) without counterpoise correction. The optimized energies, E, are in E_h and the relative energies, E_{rel}, are in cm^{-1}. Distances between nitrogen and oxygen atoms are in Å. Labeling of N and O atoms follows the notation in Fig. 3 of the main text.

	**vacuum**							
	ωB97xd/aug-cc-pVTZ				MP2/aug-cc-pVTZ			
Structure	E	E_{rel}	r($N_1 \cdot\cdot O_1$)	r($N_2 \cdot\cdot O_2$)	E	E_{rel}	r($N_1 \cdot\cdot O_1$)	r($N_2 \cdot\cdot O_2$)
**side-on**								
GG (SB)	−473.836254	0.0	2.600	2.615	−472.983285	0.0	2.556	2.618
GG ($N_1 \cdots H{-}O_1$)	−473.836520	−58.5	2.626	2.882	−472.986030	−602.3	2.614	2.883
GG ($N_2 \cdots H{-}O_2$)	−473.836595	−74.9	2.844	2.622	−472.984528	−272.7	2.829	2.619
**back-on**								
GG (SB)	−473.835104	0.0	2.593	2.611	−472.981336	0.0	2.553	2.612
GG ($N_1 \cdots H{-}O_1$)	−473.837802	−592.0	2.642	2.880	−472.986103	−1046.3	2.642	2.878
GG ($N_2 \cdots H{-}O_2$)	−473.835710	−133.0	2.854	2.625	−472.983495	−473.8	2.852	2.626

	**chloroform**							
	ωB97xd/aug-cc-pVTZ				MP2/aug-cc-pVTZ			
Structure	E(clfm)	E_{rel}(clfm)	r($N_1 \cdot\cdot O_1$)	r($N_2 \cdot\cdot O_2$)	E(clfm)	E_{rel}(clfm)	r($N_1 \cdot\cdot O_1$)	r($N_2 \cdot\cdot O_2$)
**side-on**								
GG (SB)	−473.853857	0.0	2.696	2.705	−472.999741	0.0	2.661	2.687
GG ($N_1 \cdots H{-}O_1$)	−473.845476	1839.4	2.562	2.906	−472.994684	1109.7	2.562	2.904
GG ($N_2 \cdots H{-}O_2$)	−473.845914	1743.4	2.881	2.567	−472.993531	1362.9	2.860	2.564
**back-on**								
GG (SB)	−473.853544	0.0	2.690	2.705	−472.998772	0.0	2.666	2.685
GG ($N_1 \cdots H{-}O_1$)	−473.847123	1409.2	2.592	2.916	−472.995022	823.0	2.594	2.909
GG ($N_2 \cdots H{-}O_2$)	−473.845544	1755.7	2.889	2.566	−472.992946	1278.6	2.881	2.571
